# Supplementary material for: Wide-range lifetime-tunable and responsive ultralong organic phosphorescent multi-host/guest system
Source: Nat Commun. 2021 Jun 10;12:3522. doi: 10.1038/s41467-021-23742-4 (PMC8192513; doi:10.1038/s41467-021-23742-4)
Supplement: Supplementary file 1 — Supplementary Information [file 41467_2021_23742_MOESM1_ESM.docx]

**Supplementary Information**

**Wide-range Lifetime-tunable and Responsive Ultralong Organic Phosphorescent multi-Host/Guest System**

Zongliang Xie^1^, Xiayu Zhang^2^, Hailan Wang^1^, Cheng Huang^1^, Haodong Sun^1^, Mengyang Dong^1^, Lei Ji^1^, Zhongfu An^3^, Tao Yu^1,^*, Wei Huang^1,3,4,^*

^1^ Frontiers Science Center for Flexible Electronics (FSCFE), Shaanxi Institute of Flexible Electronics (SIFE) & Shaanxi Institute of Biomedical Materials and Engineering (SIBME), Northwestern Polytechnical University (NPU), 127 West Youyi Road, Xi'an 710072, China.

^2^School of Packaging and Materials Engineering, Hunan University of Technology, Zhuzhou 412007, P. R. China

^3^Key Laboratory of Flexible Electronics (KLOFE) & Institute of Advanced Materials (IAM), Nanjing Tech University (NanjingTech), 30 South Puzhu Road, Nanjing 211816, China.

^4^State Key Laboratory of Organic Electronics and Information Displays & Jiangsu Key Laboratory for Biosensors, Institute of Advanced Materials (IAM), Nanjing University of Posts and Telecommunications, 9 Wenyuan Road, Nanjing 210023, China.

**Supplementary Figure**


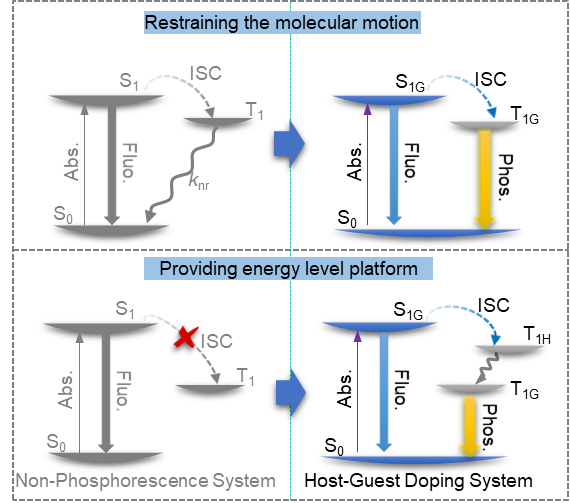


Supplementary Figure 1. Jablonski diagrams for photophysical processes in non-phosphorescence system and host/guest doping system (Abs., Fluo. and Phos. are referred to Excitation, Fluorescence and Phosphorescence).


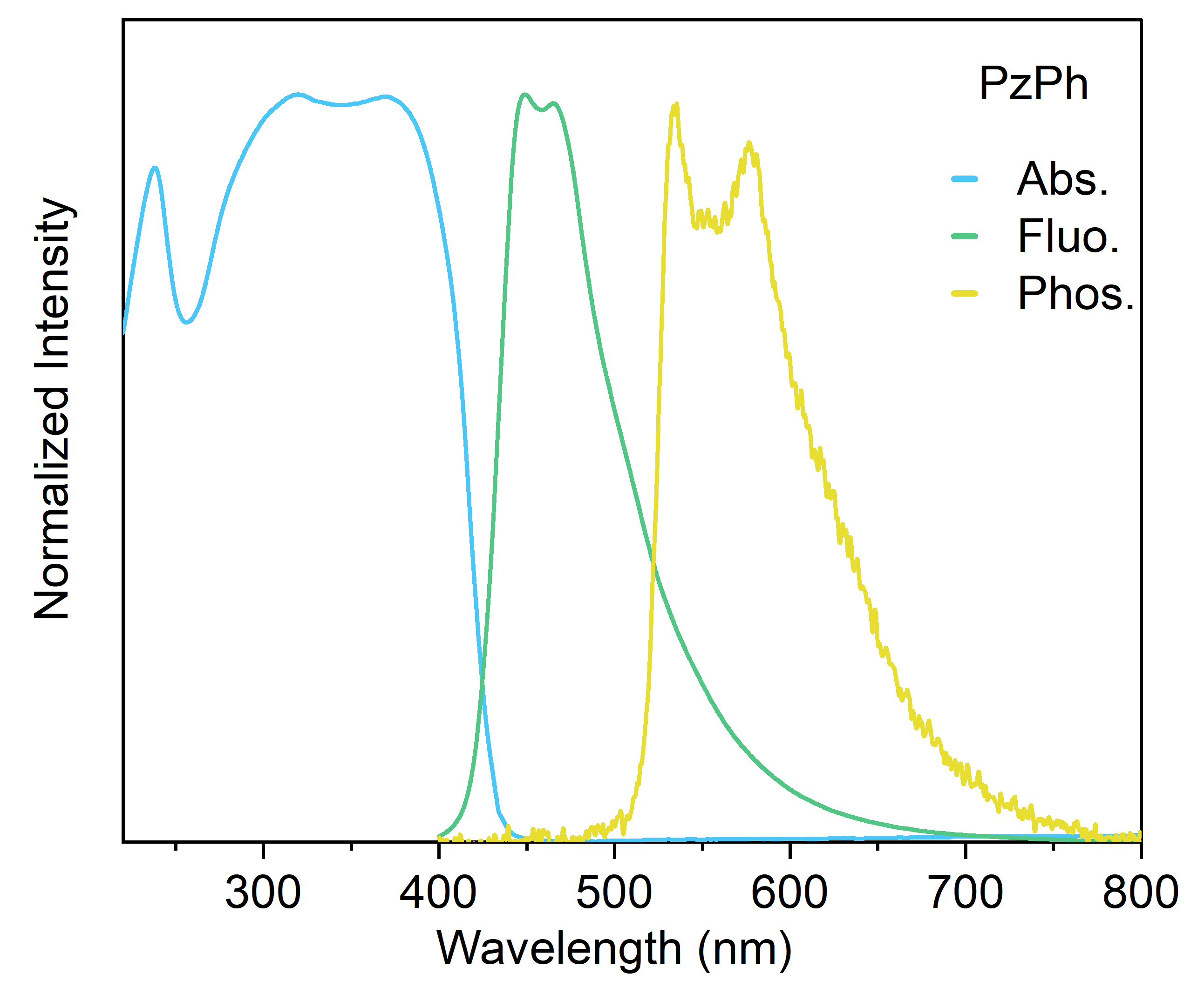


Supplementary Figure 2. Normalized absorption, fluorescence and phosphorescence spectra of the crystalline powders of PzPh. (phosphorescence spectrum was taken at 77K)


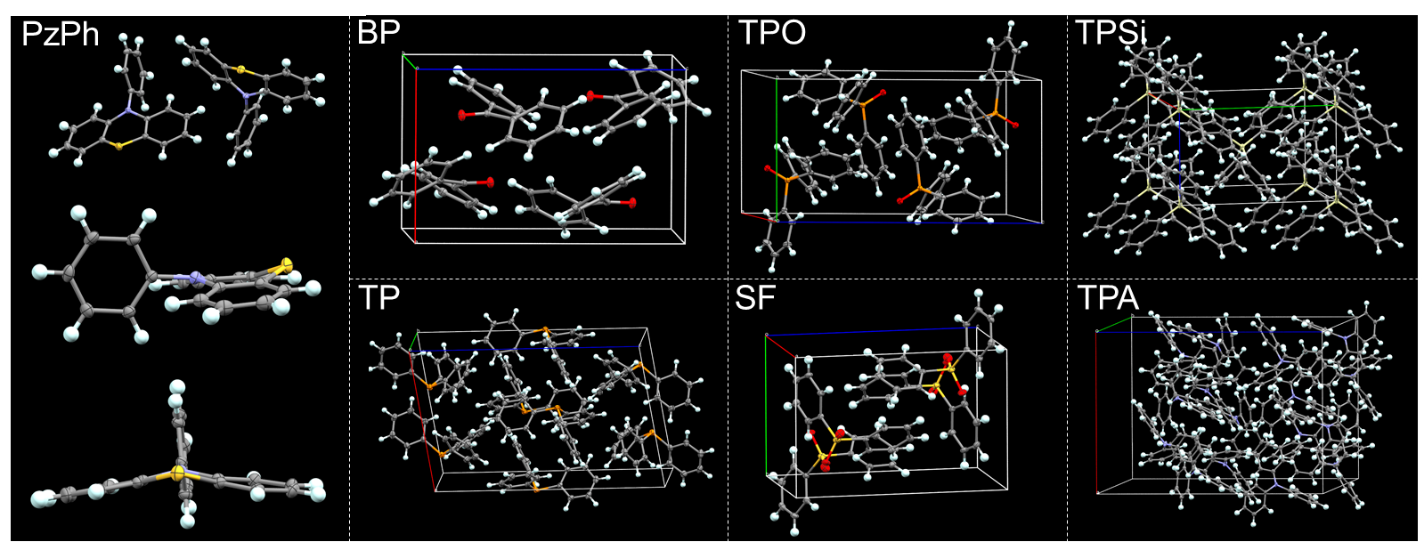


Supplementary Figure 3. Crystal structures of guest and host species (PzPh, BP, TPO, TPSi, TP, SF, TPA) and their packing modes. All the crystal structure data were downloaded at the Cambridge Crystallographic Data Centre.


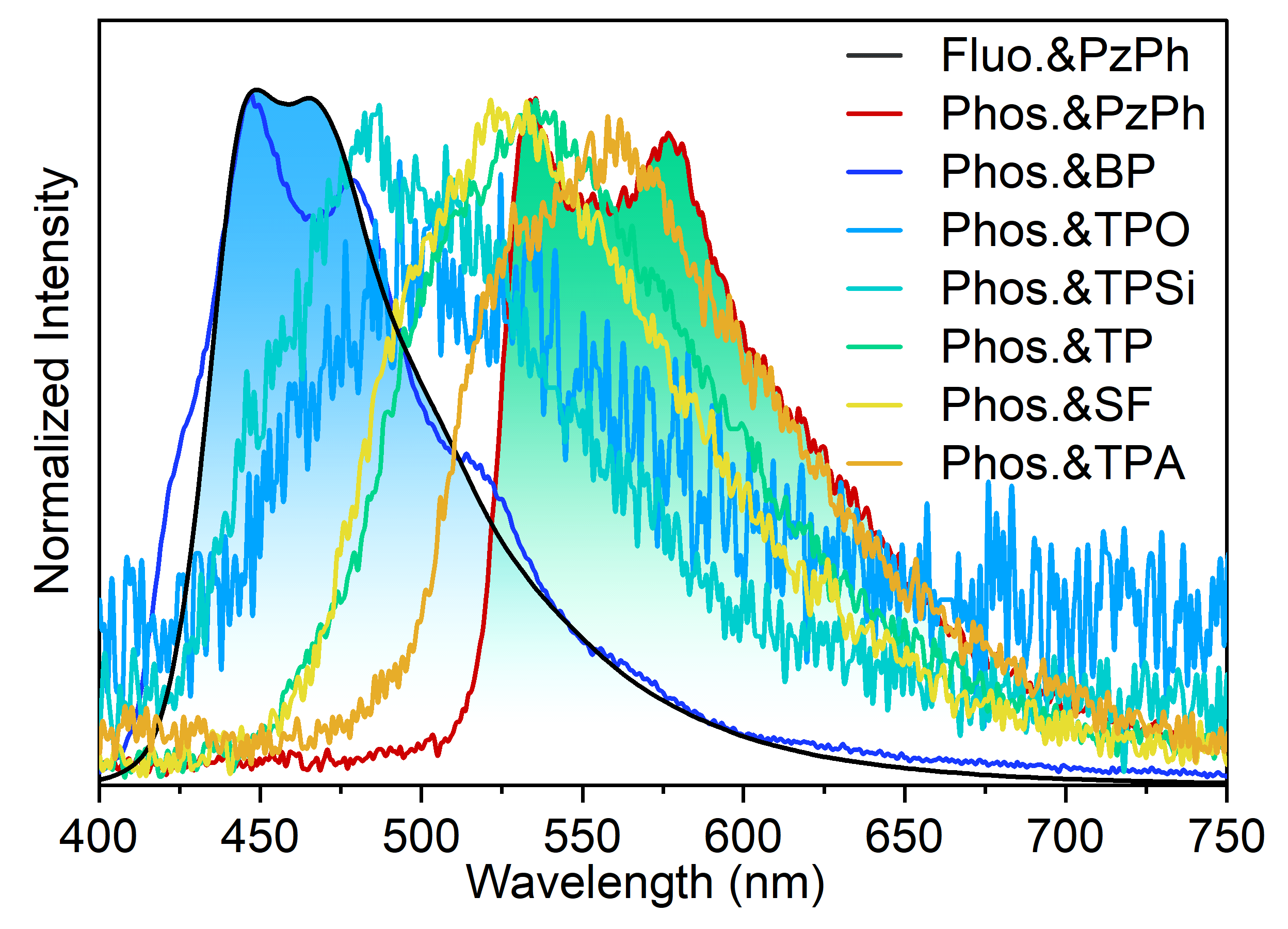


Supplementary Figure 4. Normalized phosphorescence spectra of the crystalline powders of host and guest species (PzPh, BP, TPO, TPSi, TP, SF, TPA) at 8ms after 365 nm excitation ceased. (phosphorescence spectra of TP and PzPh were taken at 77K, while others were taken at room temperature)


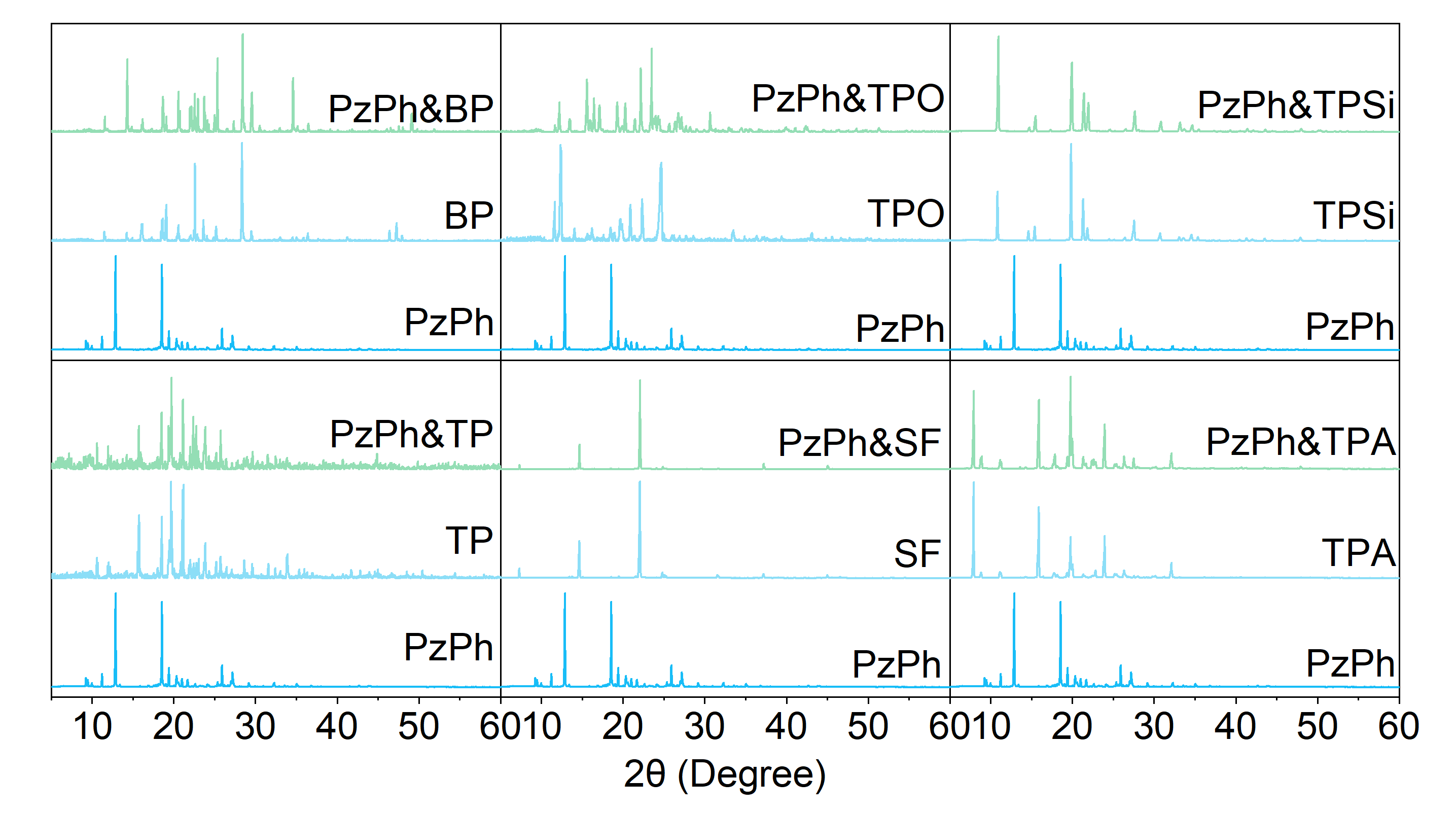


Supplementary Figure 5. Powder X-ray diffraction (PXRD) patterns of the mH/G UOP materials and their corresponding host and guest species.


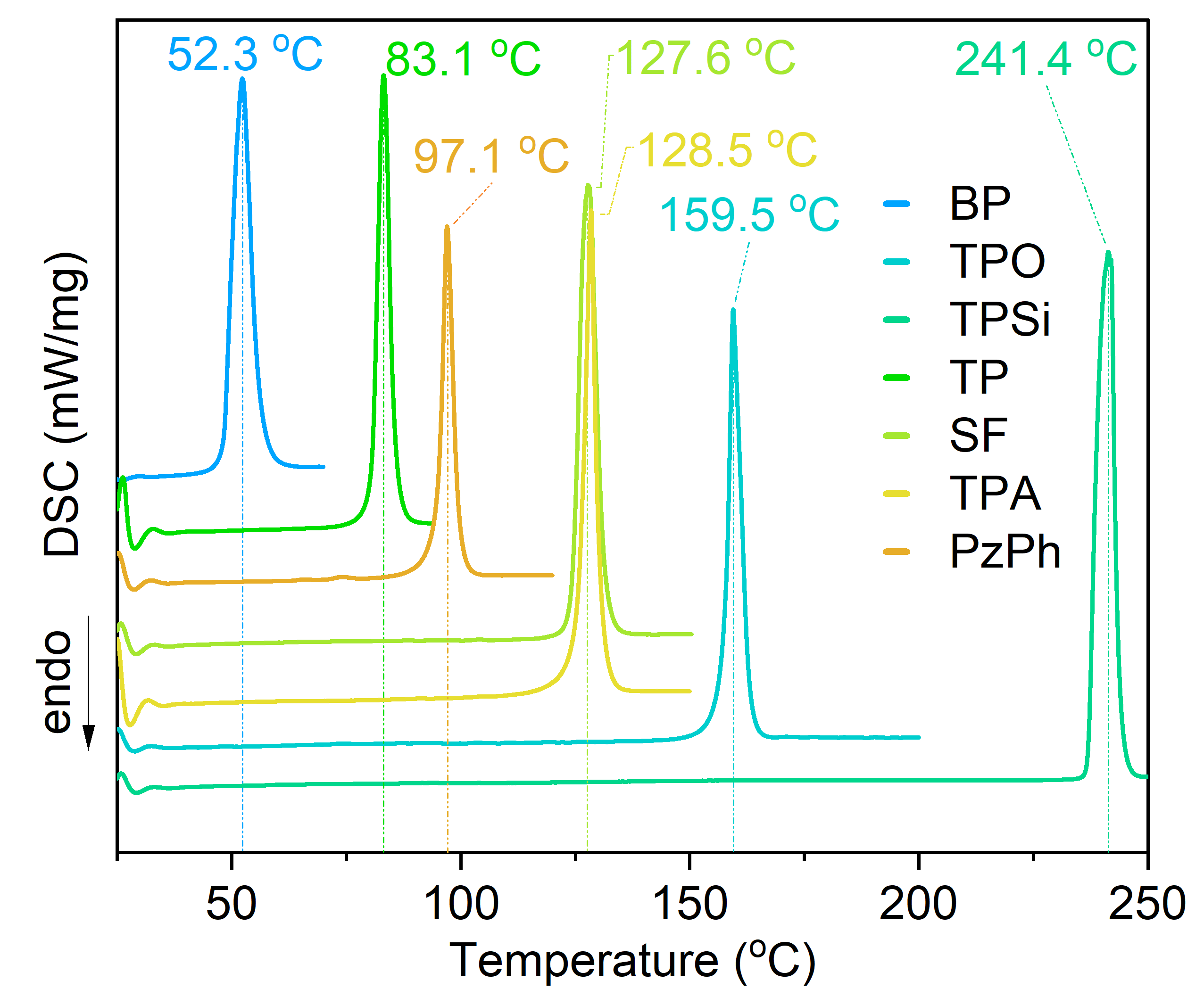


Supplementary Figure 6. Differential scanning calorimeter (DSC) curves of PzPh and host materials (BP, TPO, TPSi, TP, SF and TPA) under N_2_ atmosphere at 10 ^o^C/min heating rate.


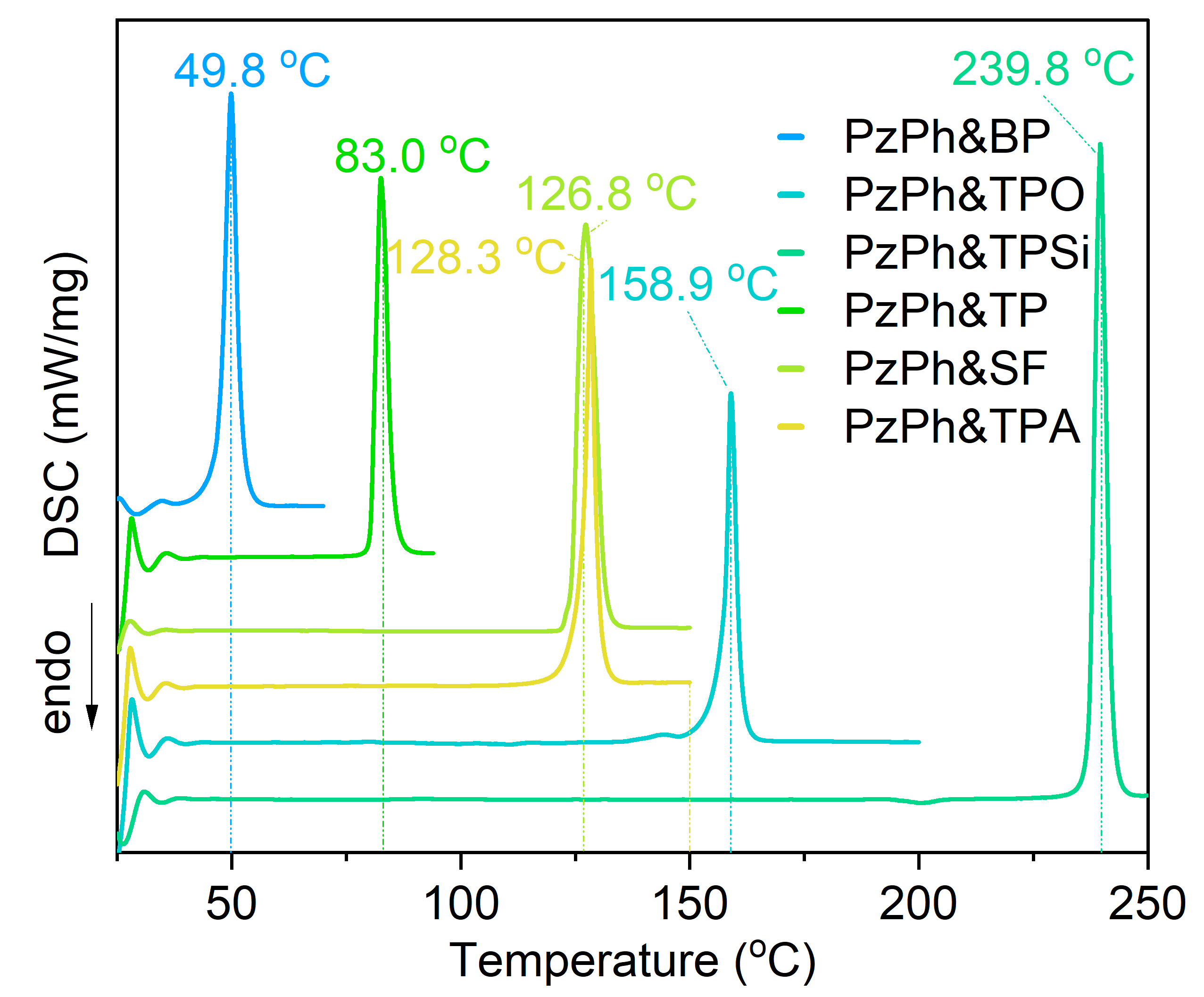


Supplementary Figure 7. DSC curves of the mH/G UOP system under N_2_ atmosphere at 10 ^o^C/min heating rate.


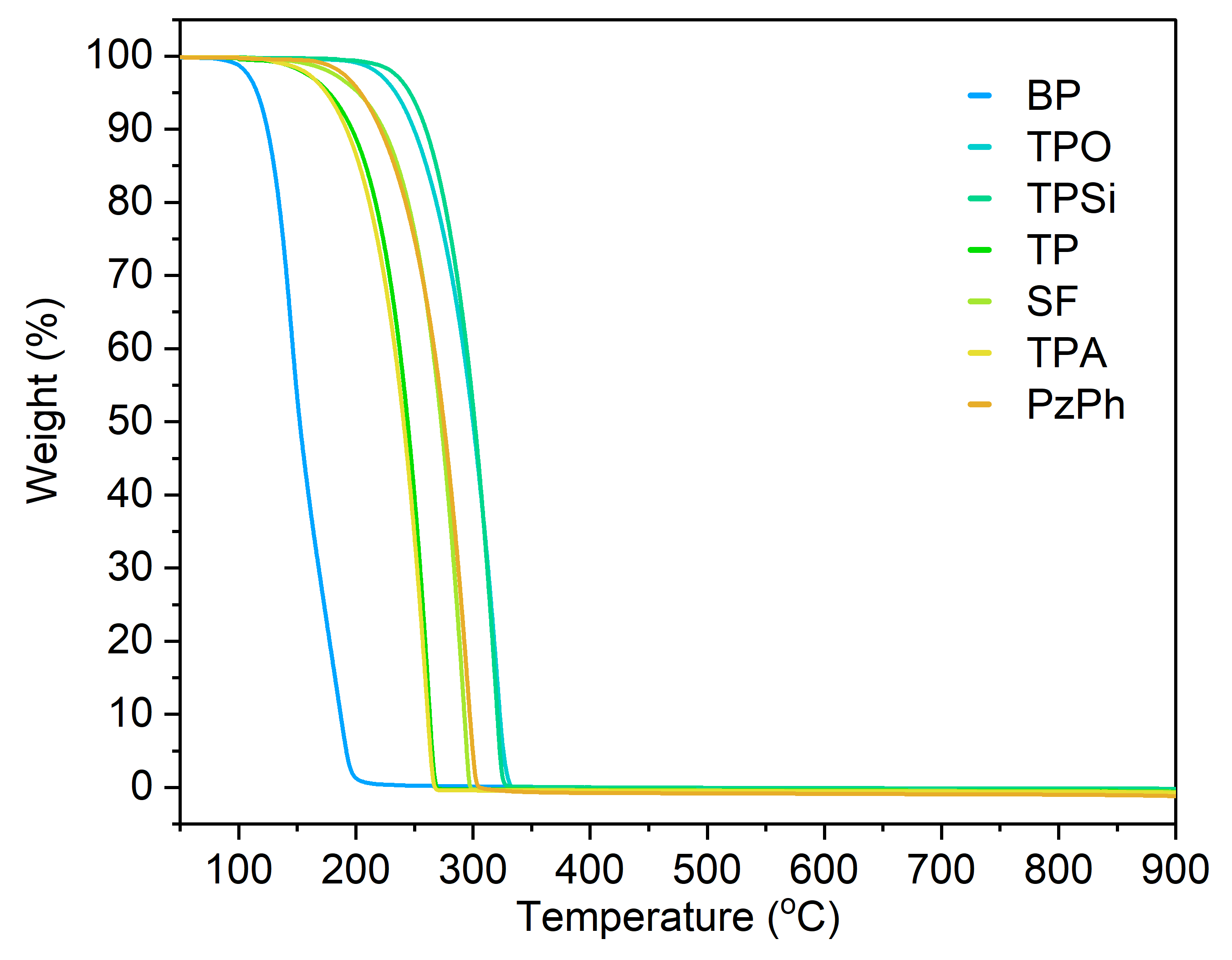


Supplementary Figure 8. Thermogravimetry analyses (TGA) curves of PzPh and host materials (BP, TPO, TPSi, TP, SF and TPA) under N_2_ atmosphere at 20 ^o^C/min heating rate.


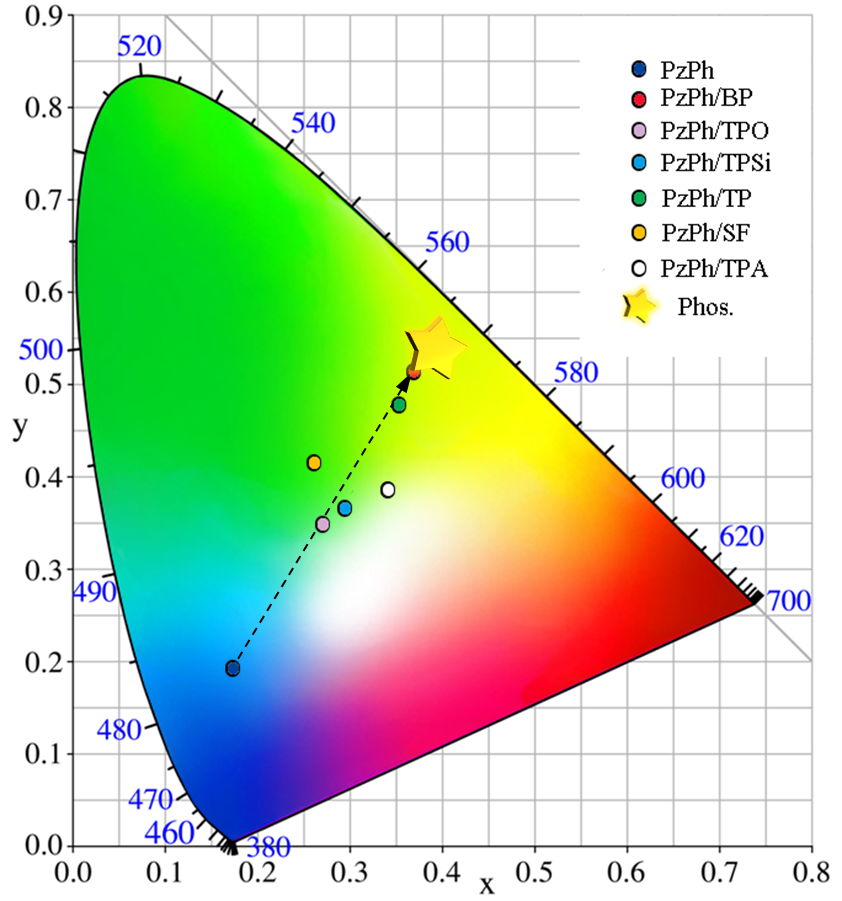


Supplementary Figure 9. Commission Internationale de L’Eclaerage (CIE) chromaticity coordinates of fluorescence and phosphorescence emission colors of crystalline powders for PzPh and mH/G UOP materials under 365 nm irradiation at room temperature. (values of the CIE coordinates were fitted from their corresponding steady-state emission spectra)


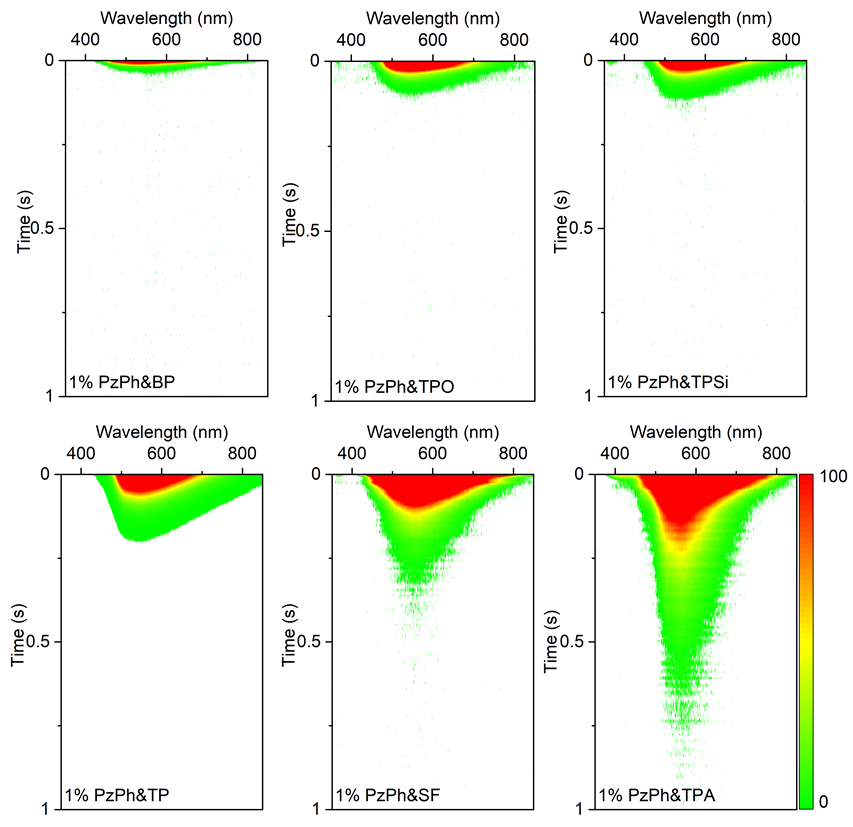


Supplementary Figure 10. Time-resolved phosphorescence spectra for the crystalline powders of the mH/G UOP system, excited at 365 nm and integral time 8 ms.


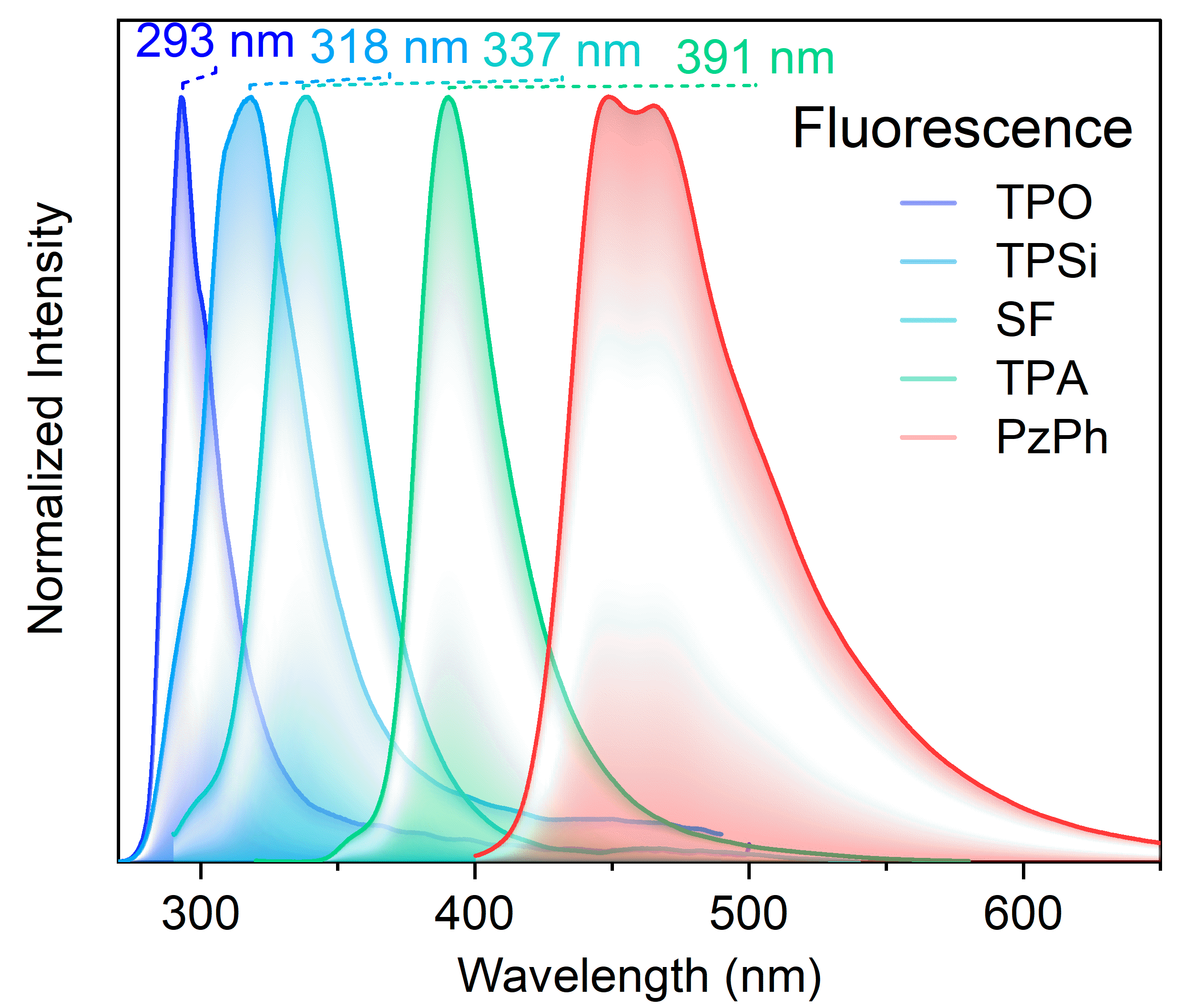


Supplementary Figure 11. Fluorescence spectra of host and guest species (PzPh, TPO, TPSi, SF, TPA).


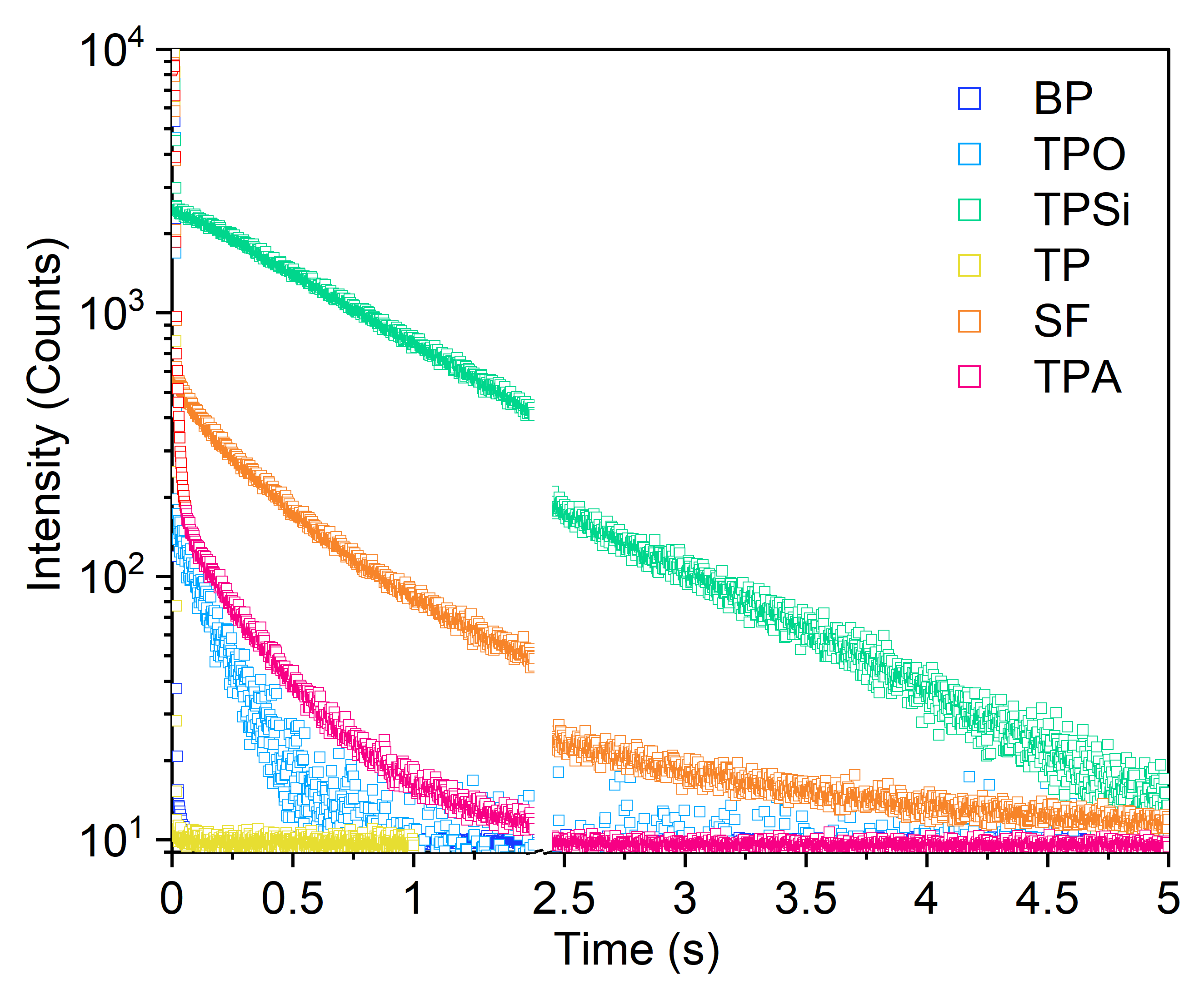


Supplementary Figure 12. Time-resolved decay curves for host materials at room temperature.


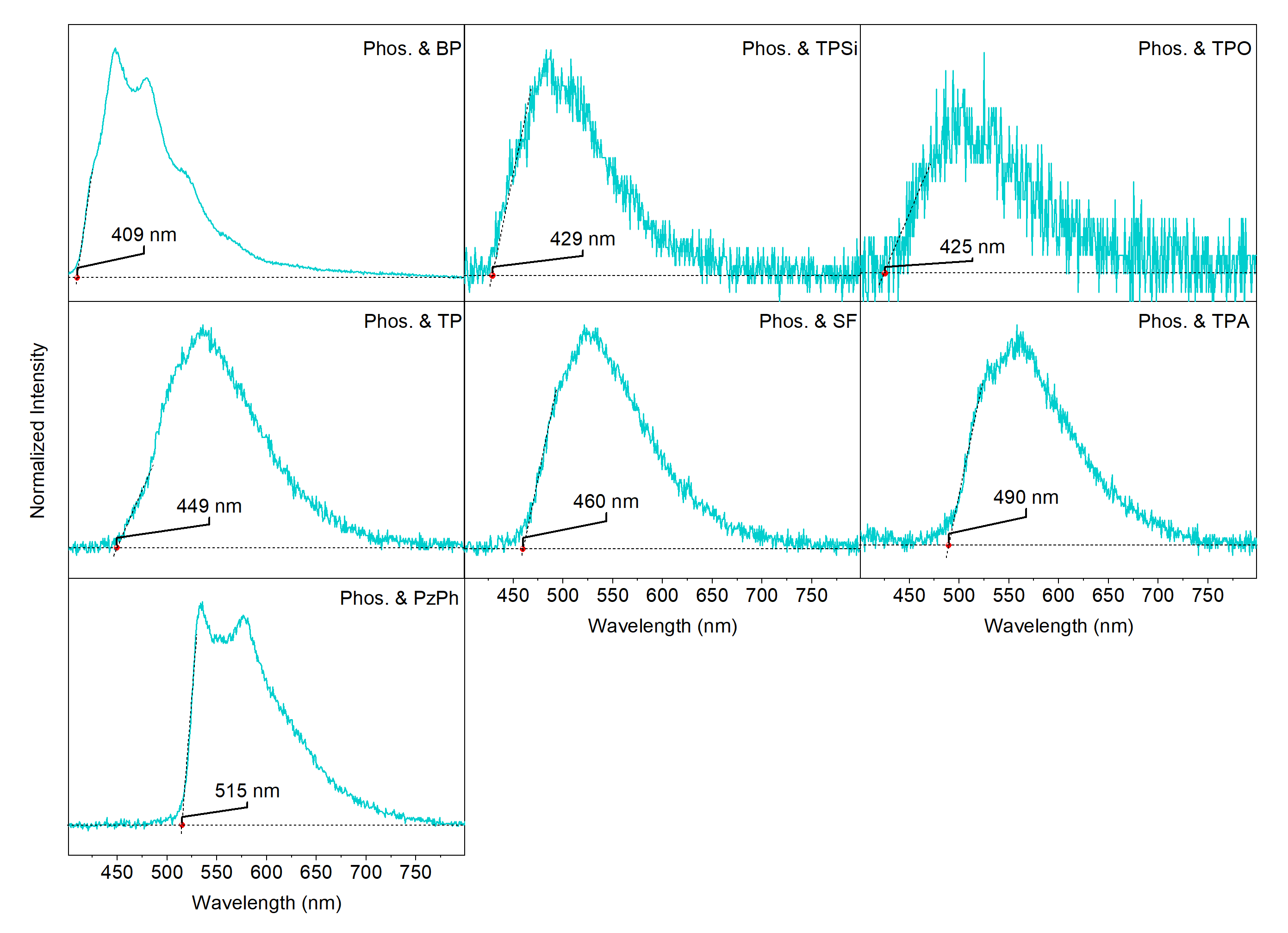


Supplementary Figure 13. Tangent line of phosphorescence spectra to calculate the triplet energy levels of host and guest materials (PzPh, BP, TPO, TPSi, TP, SF, TPA).


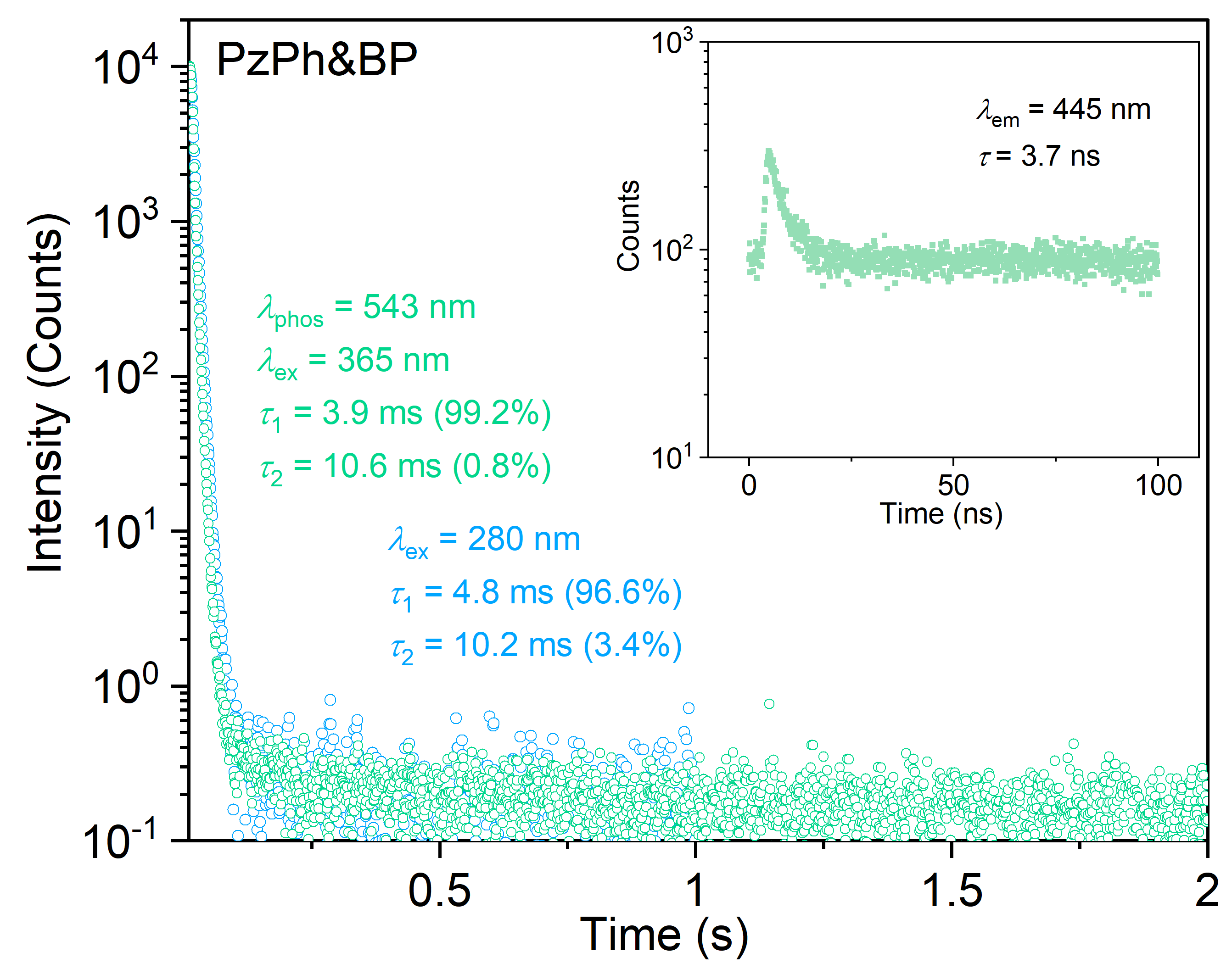


Supplementary Figure 14. Time-resolved decay curves of PzPh&BP under 365 nm and 280 nm excitation at room temperature.


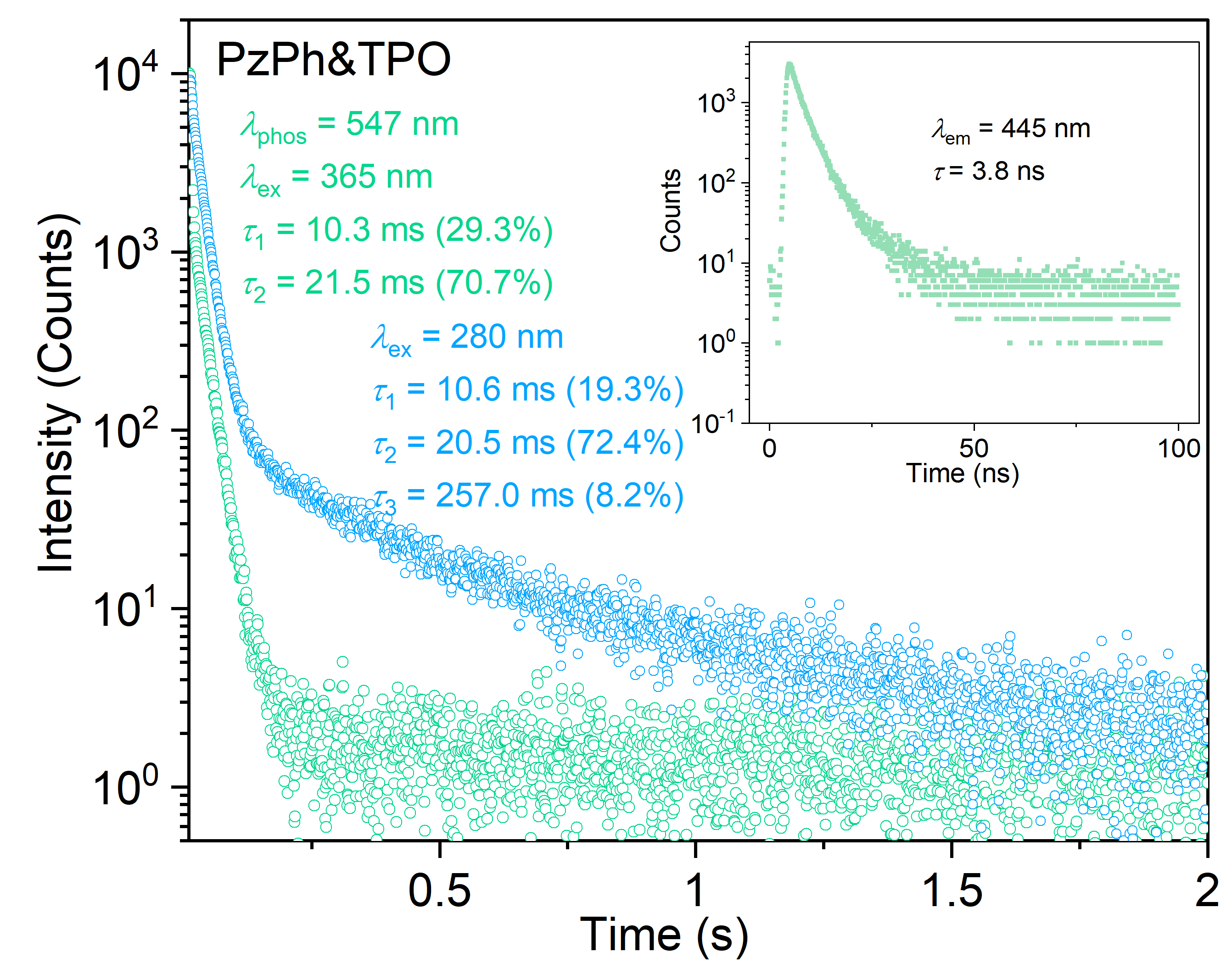


Supplementary Figure 15. Time-resolved decay curves of PzPh&TPO under 365 nm and 280 nm excitation at room temperature.


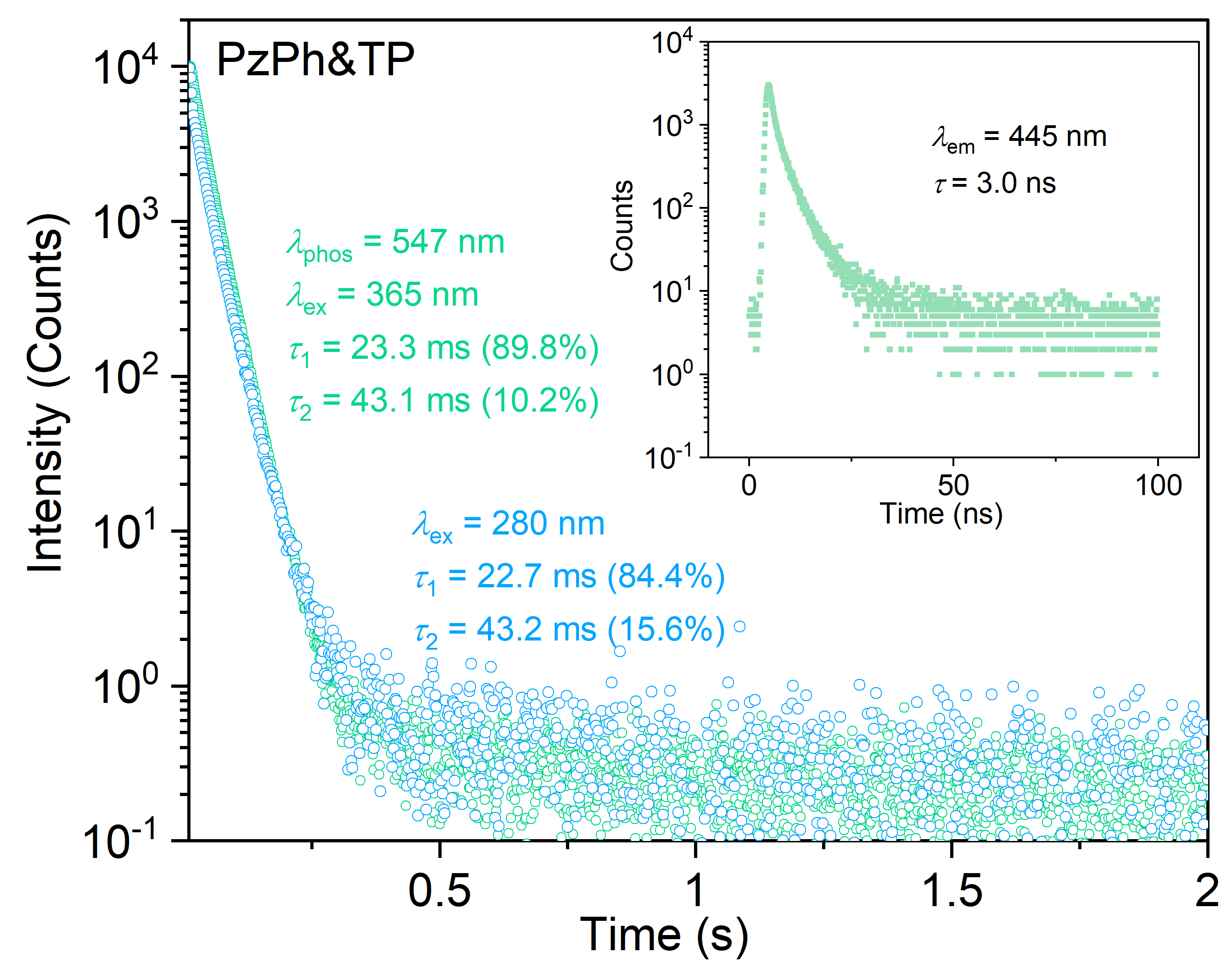


Supplementary Figure 16. Time-resolved decay curves of PzPh&TP under 365 nm and 280 nm excitation at room temperature.


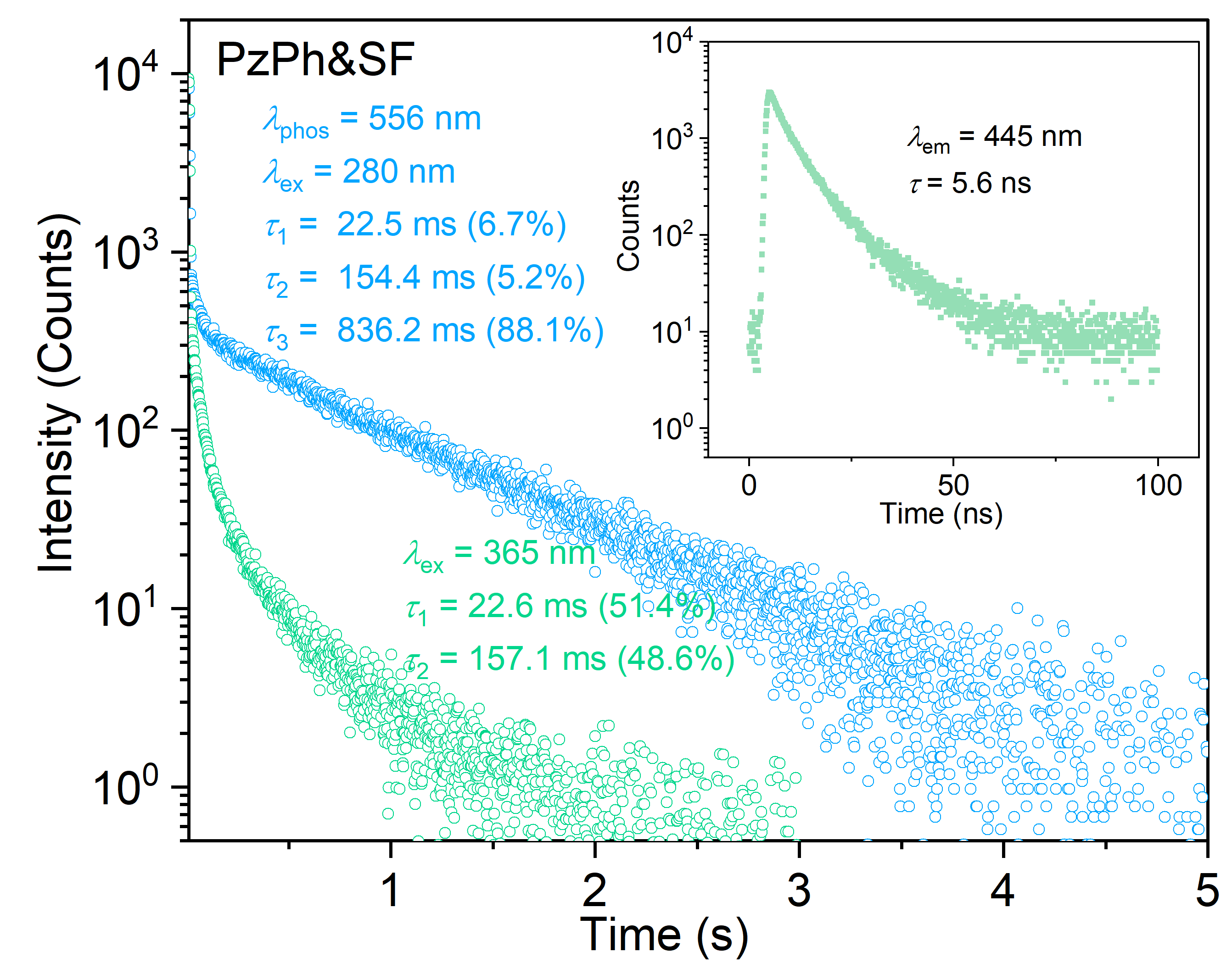


Supplementary Figure 17. Time-resolved decay curves of PzPh&SF under 365 nm and 280 nm excitation at room temperature.


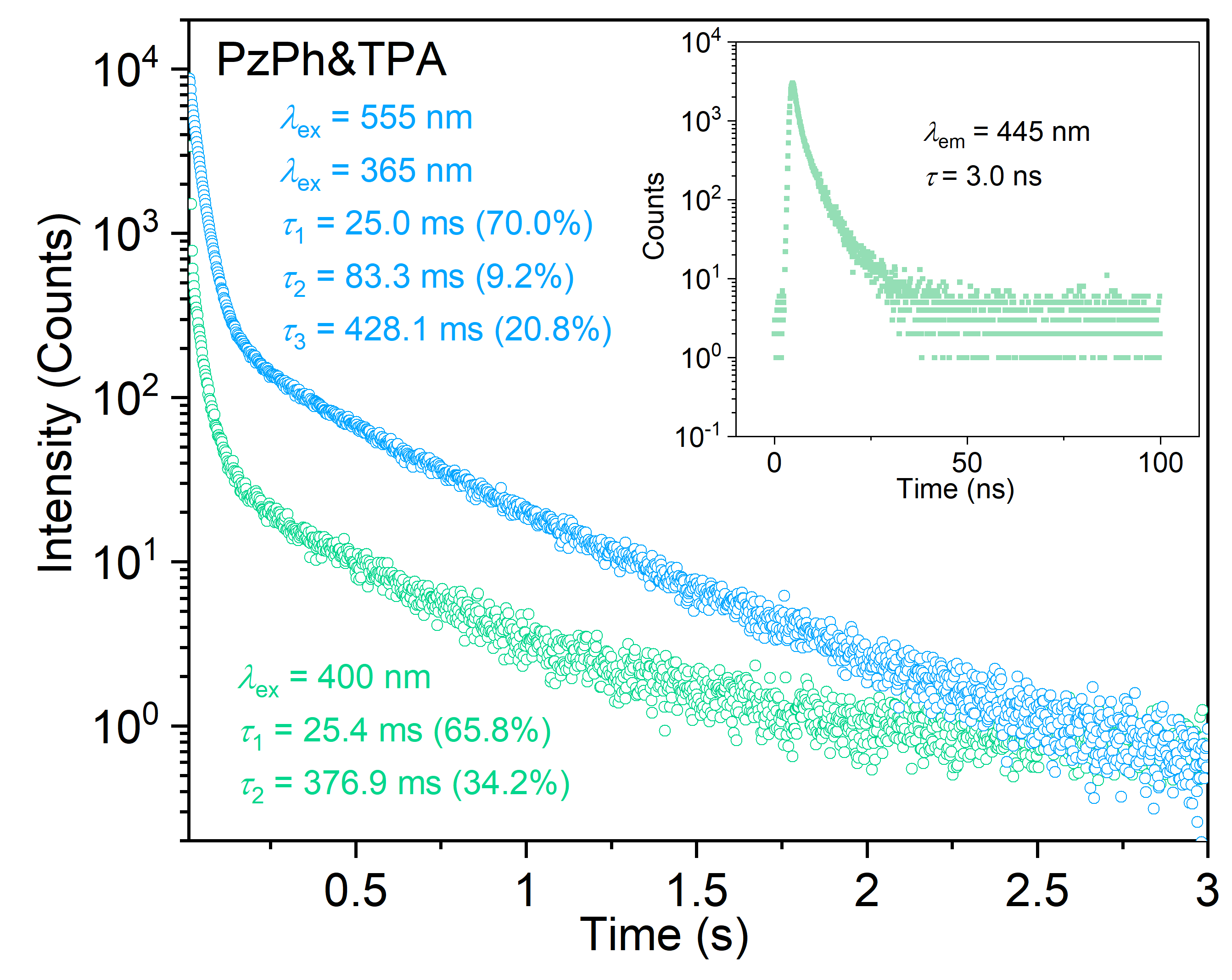


Supplementary Figure 18. Time-resolved decay curves of PzPh&TPA under 365 nm and 400 nm excitation at room temperature.


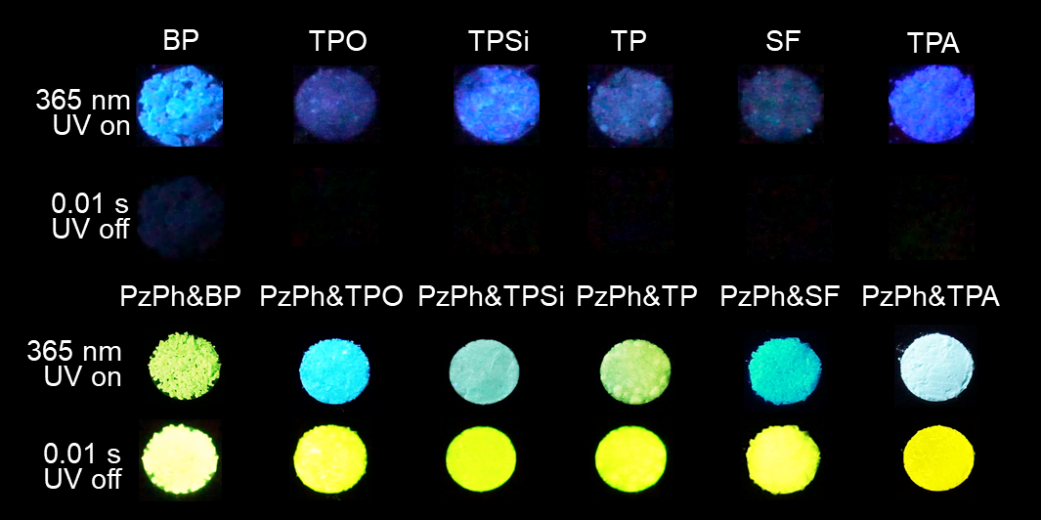


Supplementary Figure 19. Photographs of host and the mH/G UOP materials at the conditions of "365 nm UV on" and "0.01 s UV off".

**
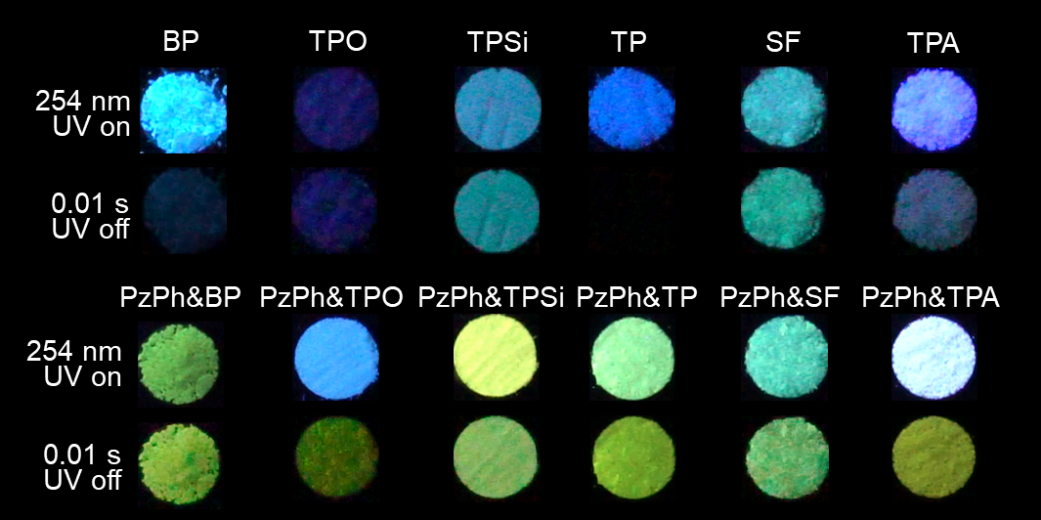
**

Supplementary Figure 20. Photographs of host and the mH/G UOP materials at the conditions of "254 nm UV on" and "0.01 s UV off".

**
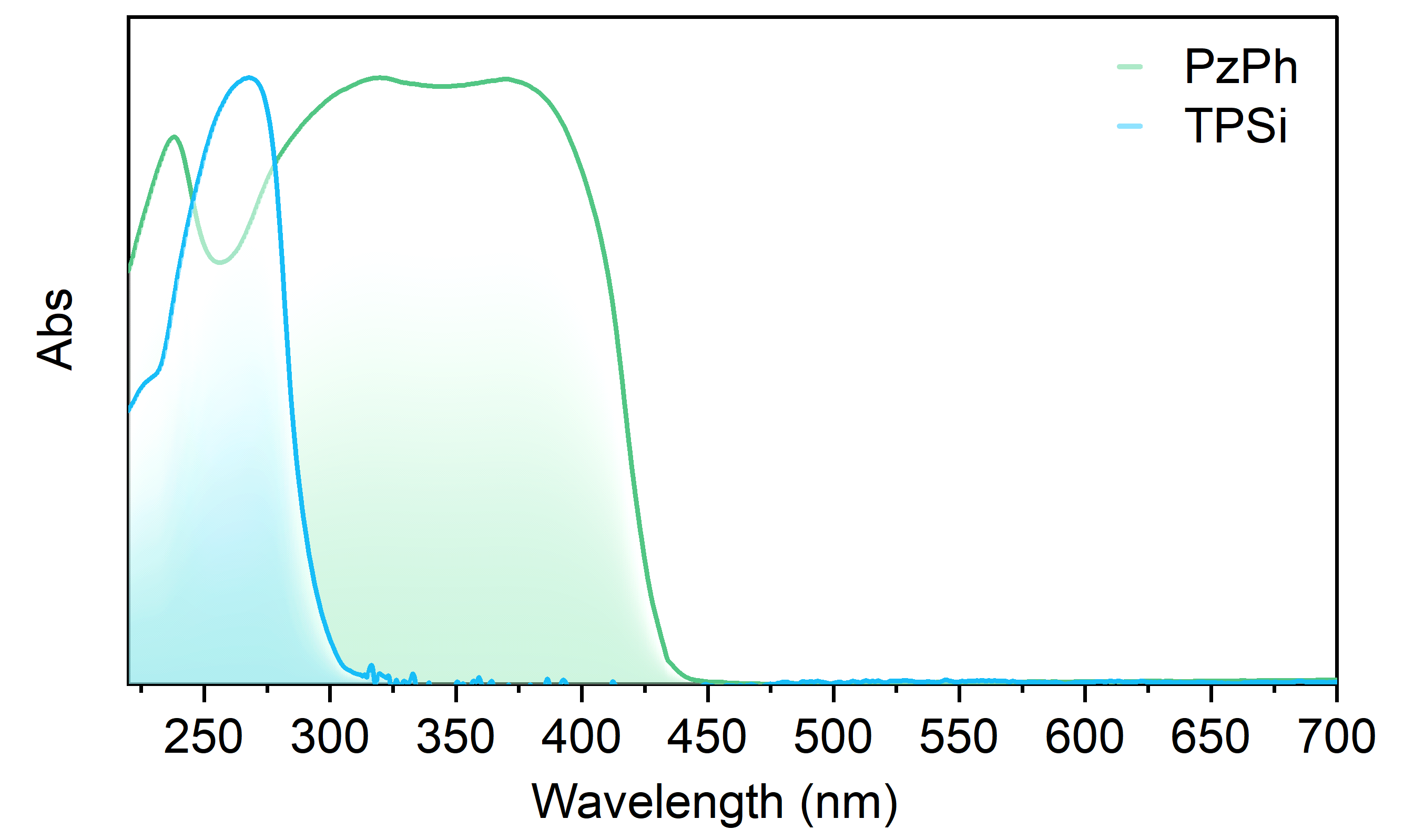
**

Supplementary Figure 21. Normalized absorption spectra of the crystalline powders for PzPh and TPSi.

**
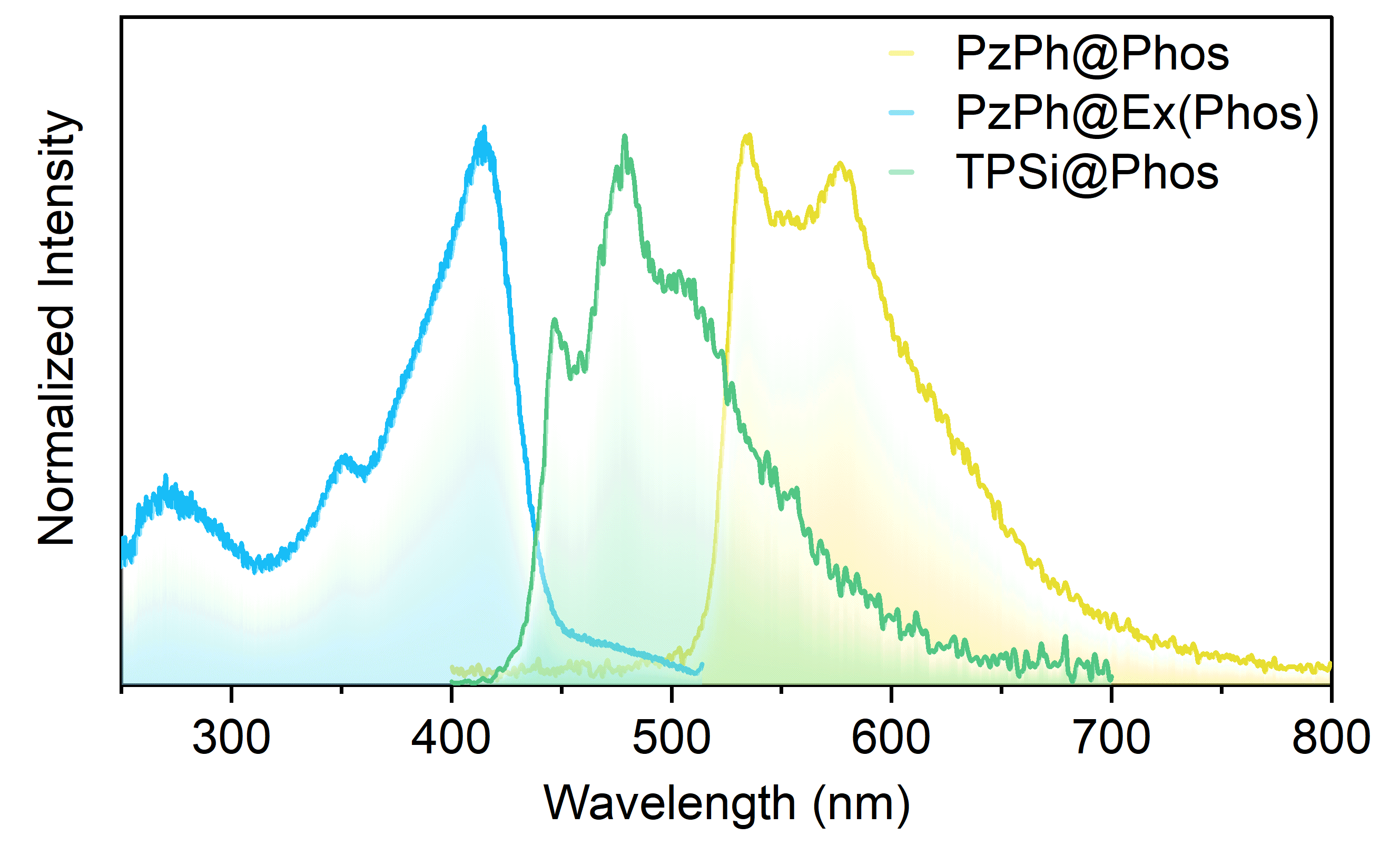
**

Supplementary Figure 22. Phosphorescence spectra of PzPh and TPSi and the excitation spectrum of PzPh at phosphorescence peak 543 nm taken at 77K.

**
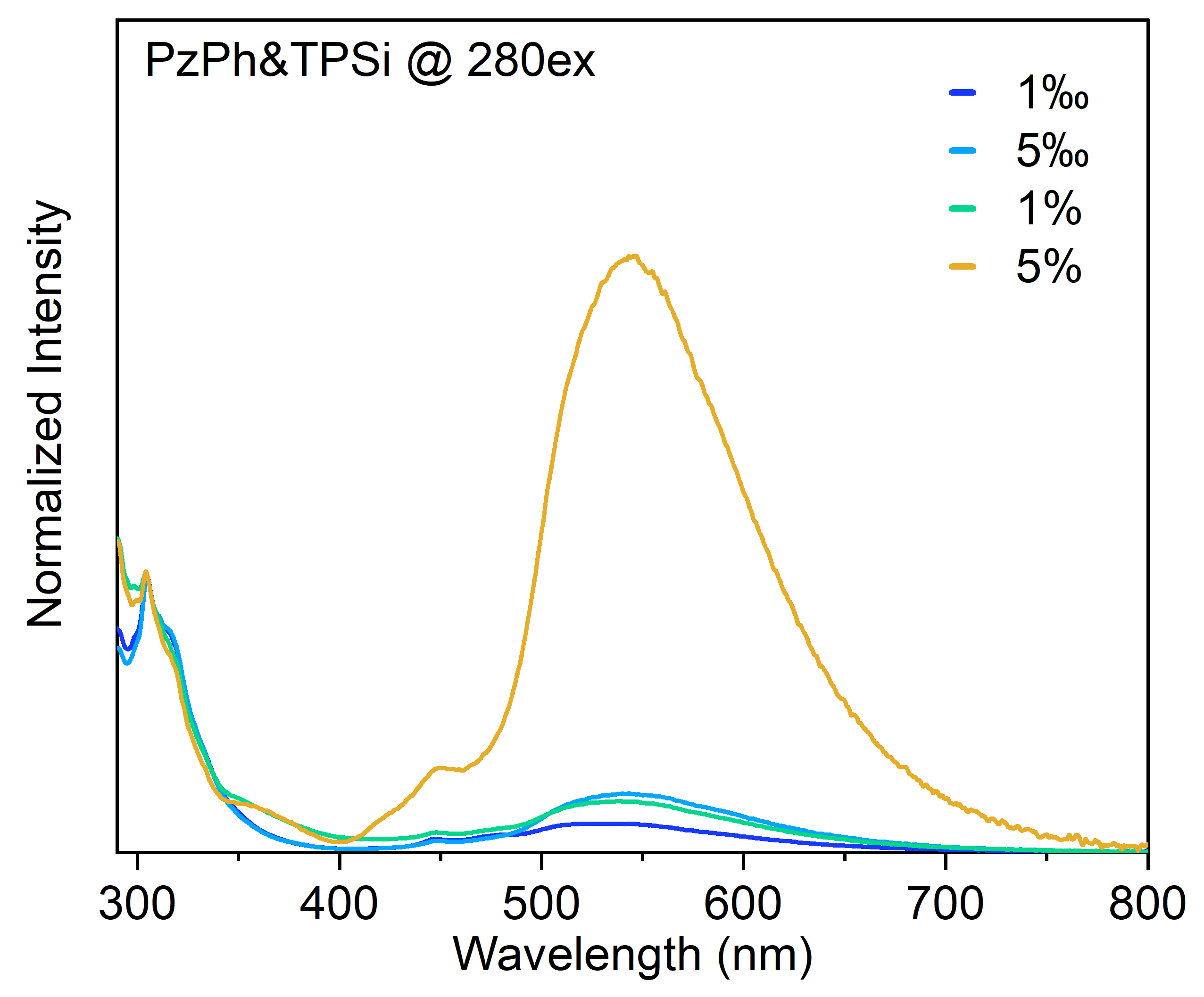
**

Supplementary Figure 23. Photoluminescence spectra of PzPh&TPSi under 280 nm excitation with different concentration (to facilitate comparison, the spectra were normalized at 304 nm).

**
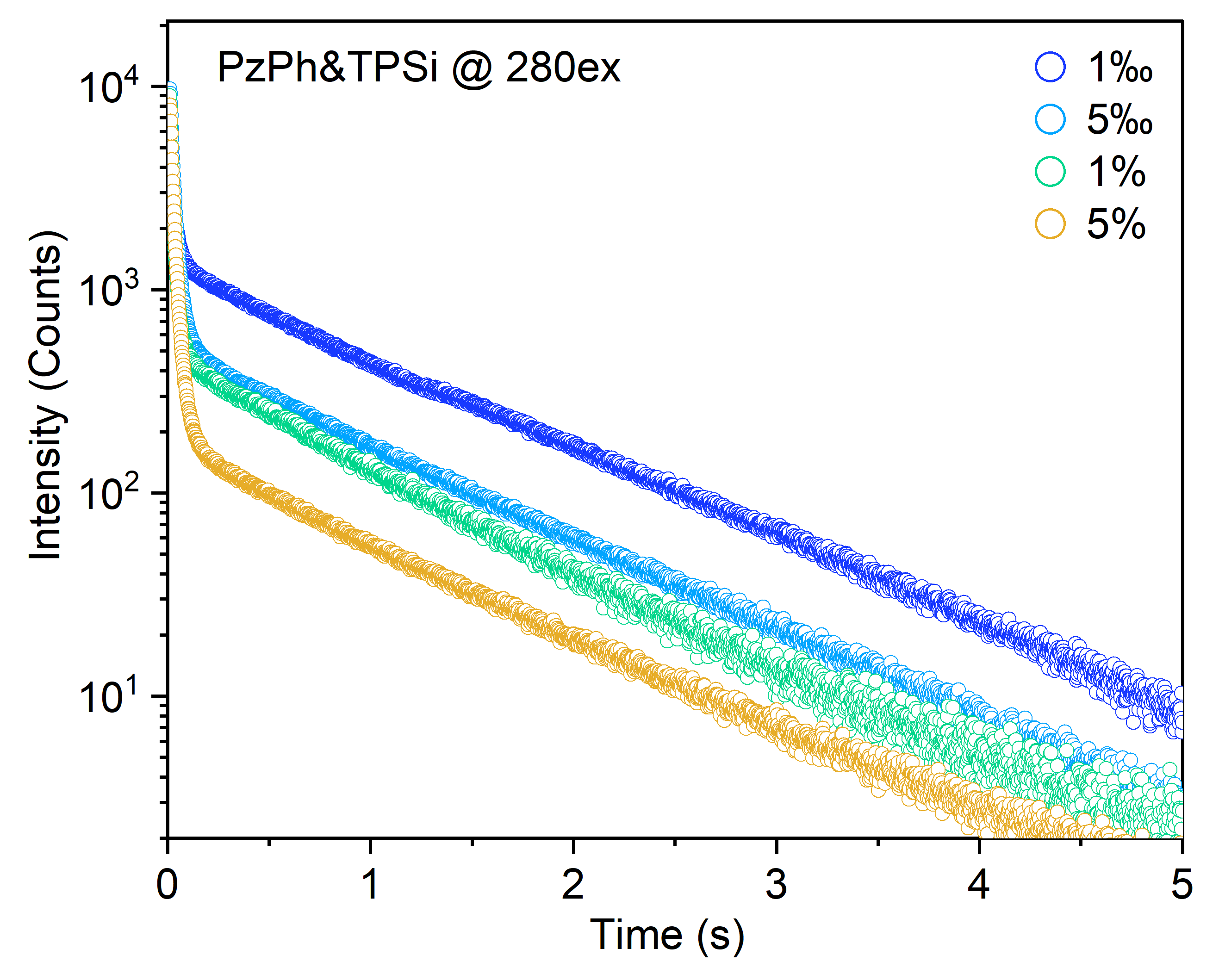
**

Supplementary Figure 24. Time-resolved decay curves of PzPh&TPSi at 549 nm under 280 nm excitation with different concentration.


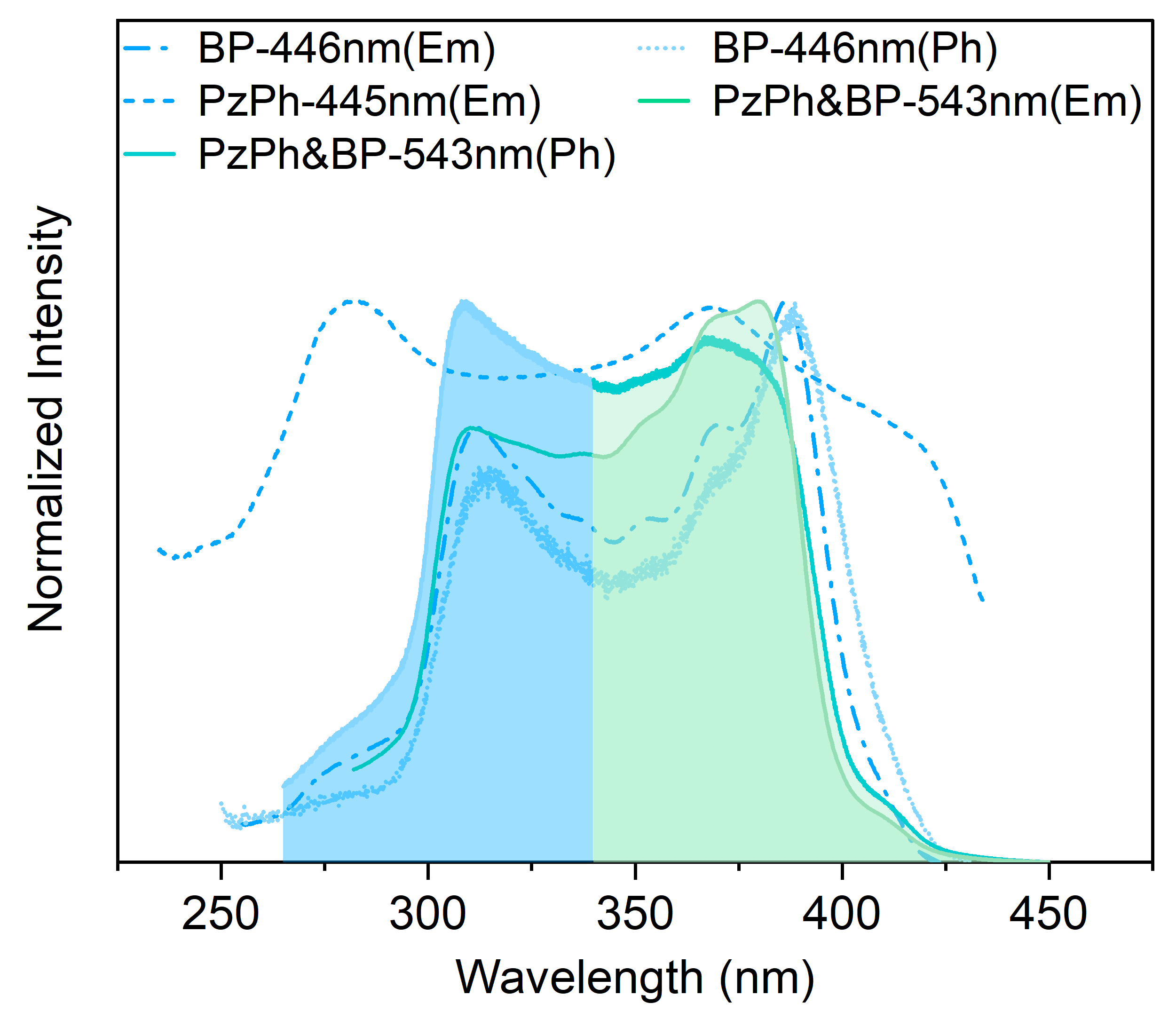


Supplementary Figure 25. Excitation spectra of PzPh, BP, and their doping system (“Em” was referred to the steady-state emission peak, and “Ph” was referred to the phosphorescence emission peak).


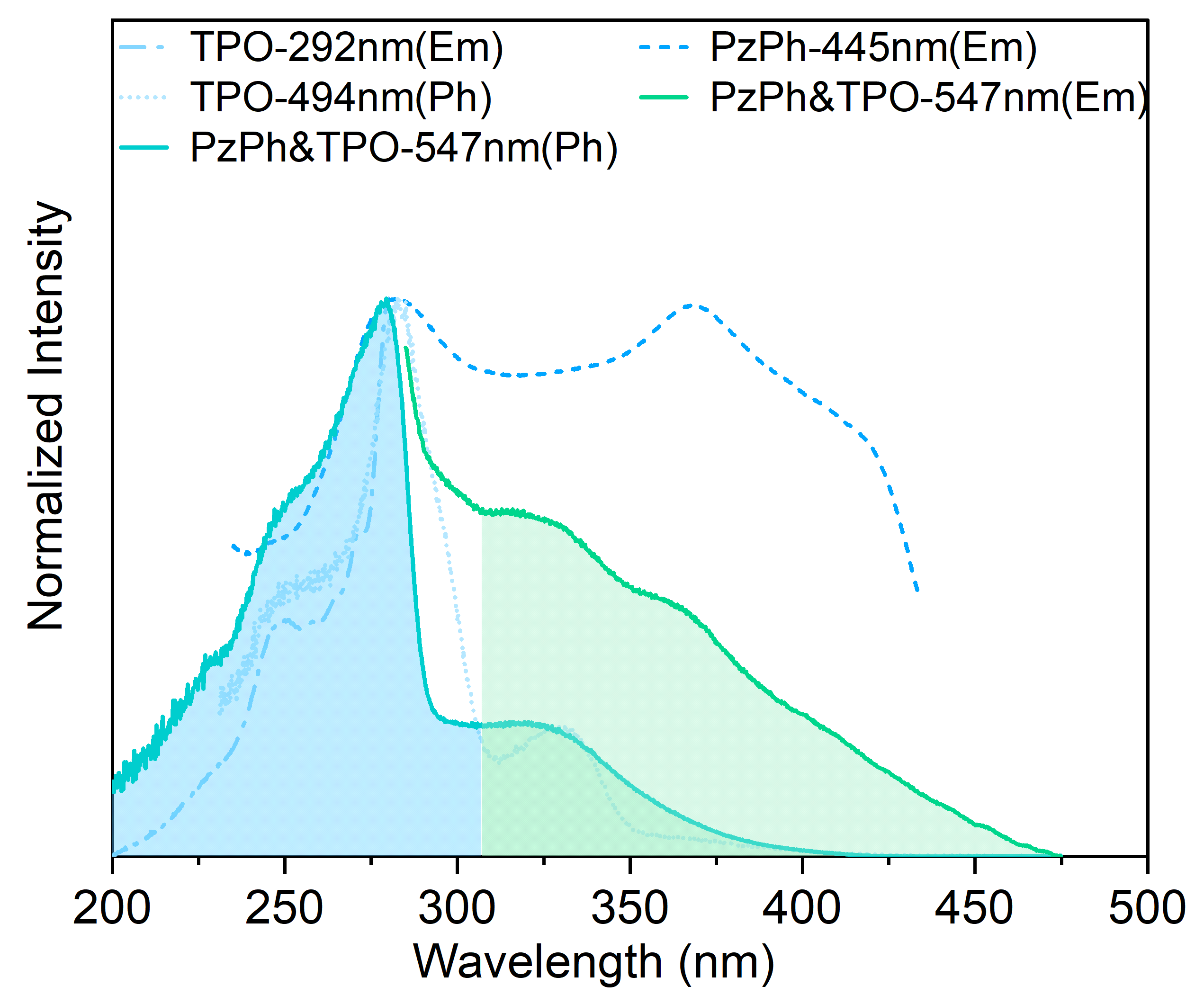


Supplementary Figure 26. Excitation spectra of PzPh, TPO, and their doping system.


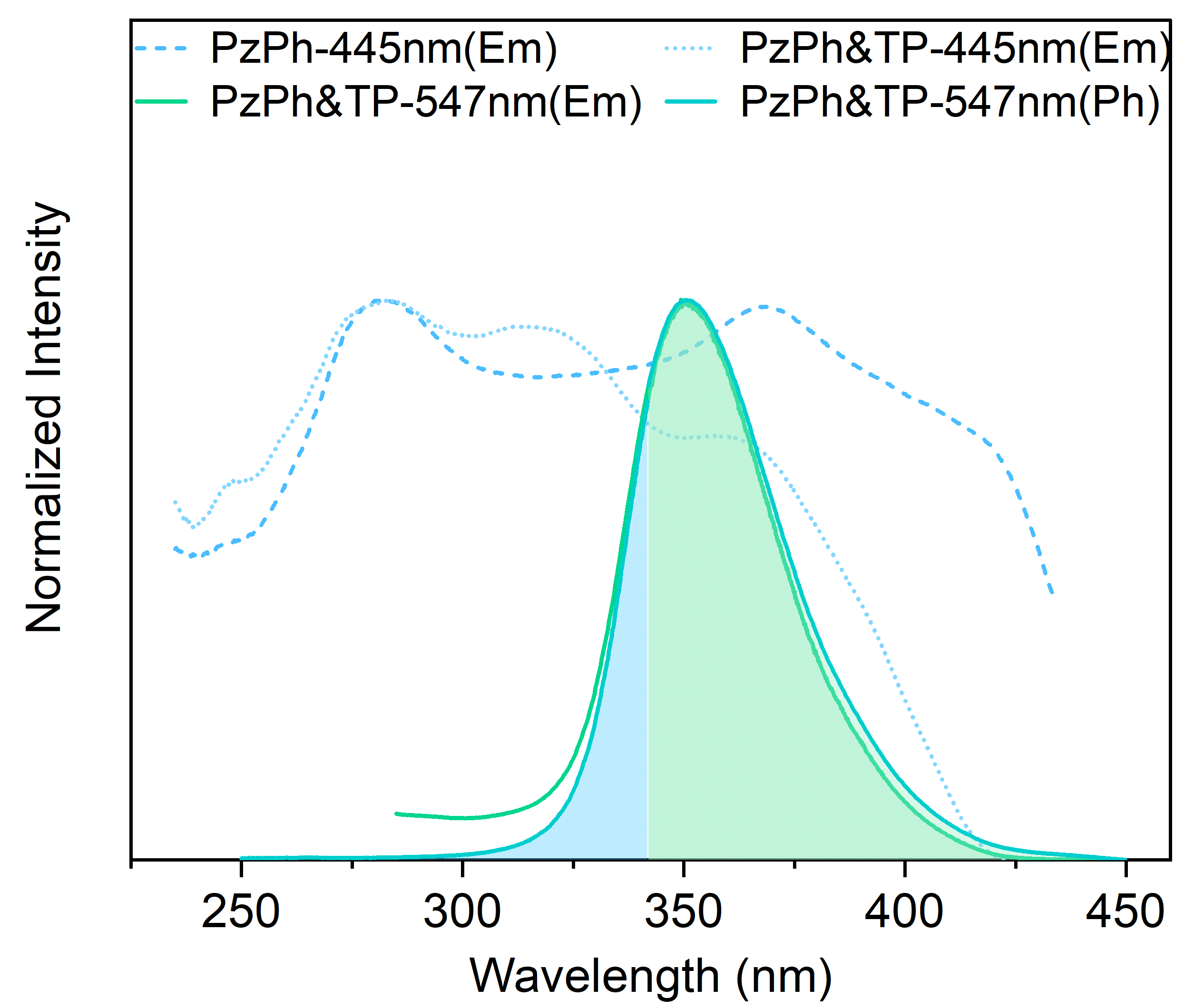


Supplementary Figure 27. Excitation spectra of PzPh, TP, and their doping system.


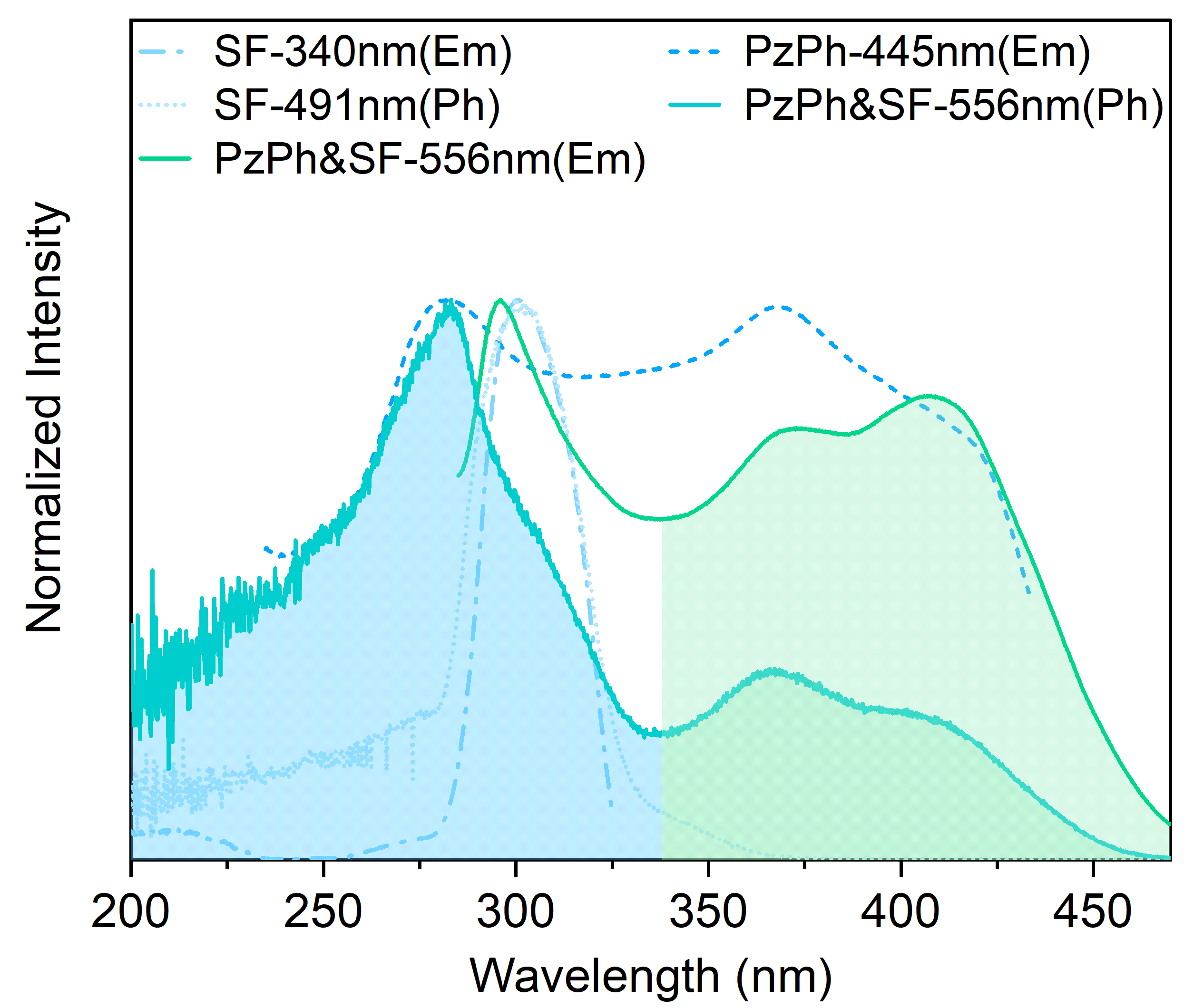


Supplementary Figure 28. Excitation spectra of PzPh, SF, and their doping system.


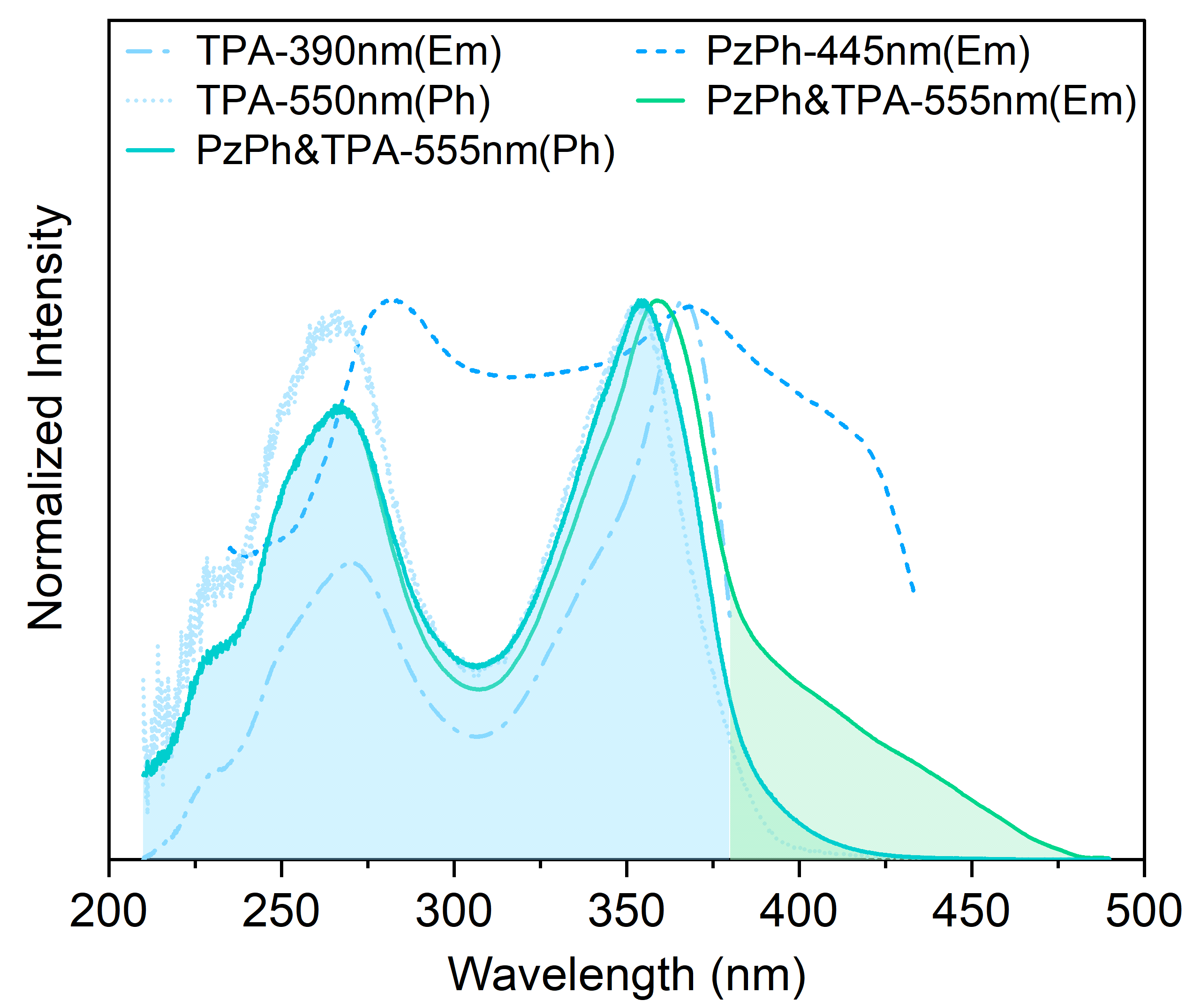


Supplementary Figure 29. Excitation spectra of PzPh, TPA, and their doping system.


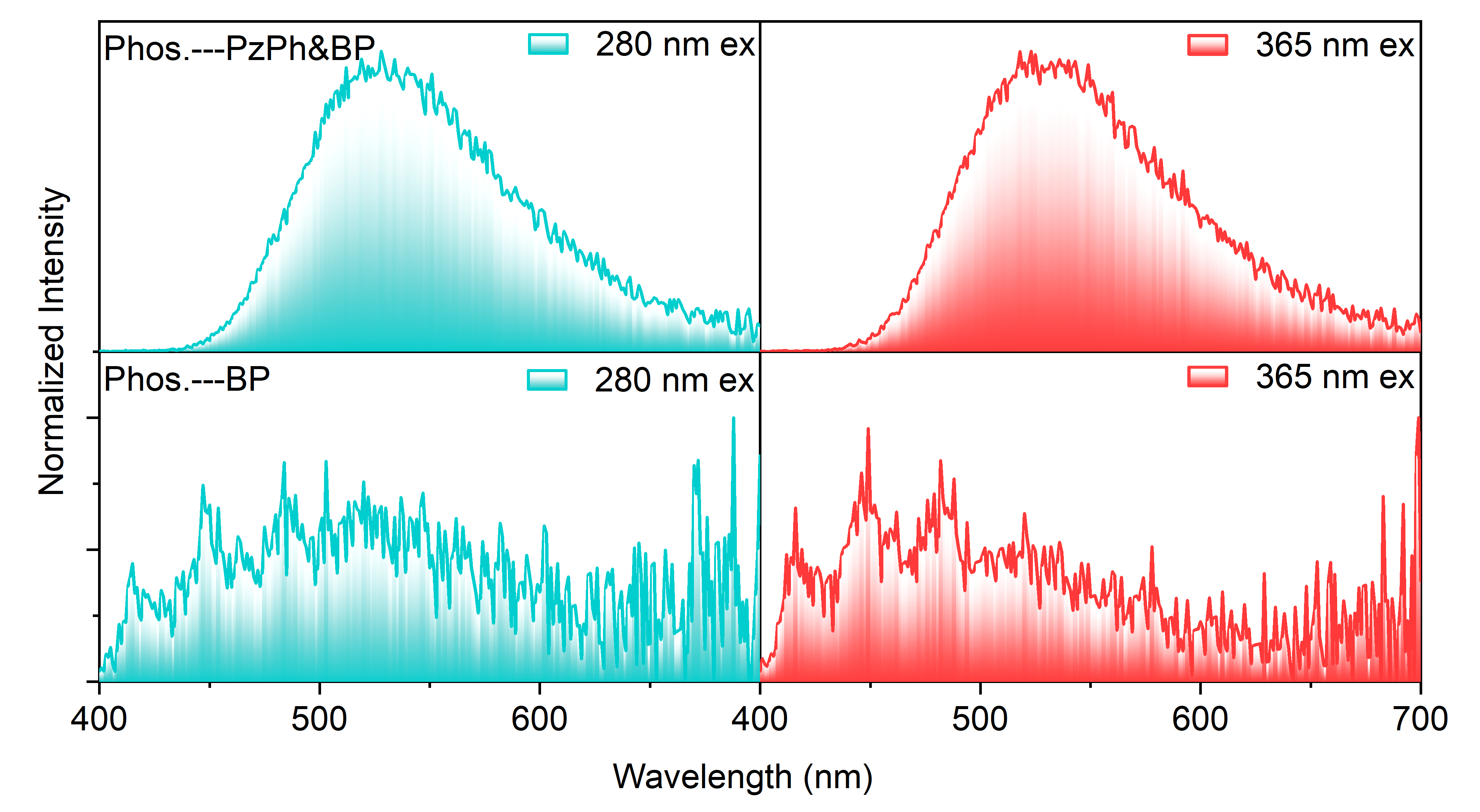


Supplementary Figure 30. Normalized delay emission spectra of PzPh&BP and BP after the stoppage of different excitation wavelengths (virtual gating from 1 ms to 5 ms).


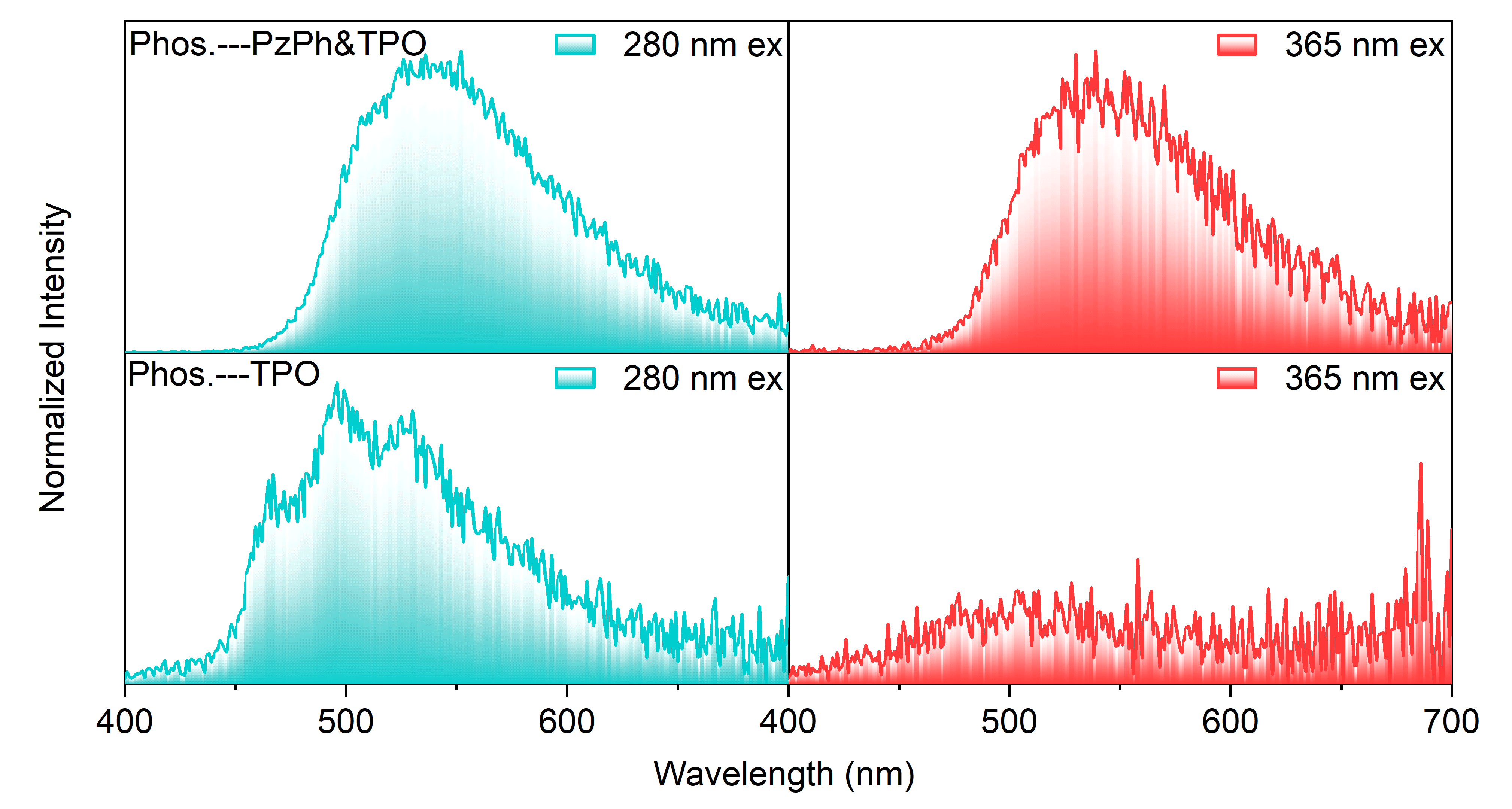


Supplementary Figure 31. Normalized delay emission spectra of PzPh&TPO and TPO after the stoppage of different excitation wavelengths (virtual gating from 1 ms to 5 ms).


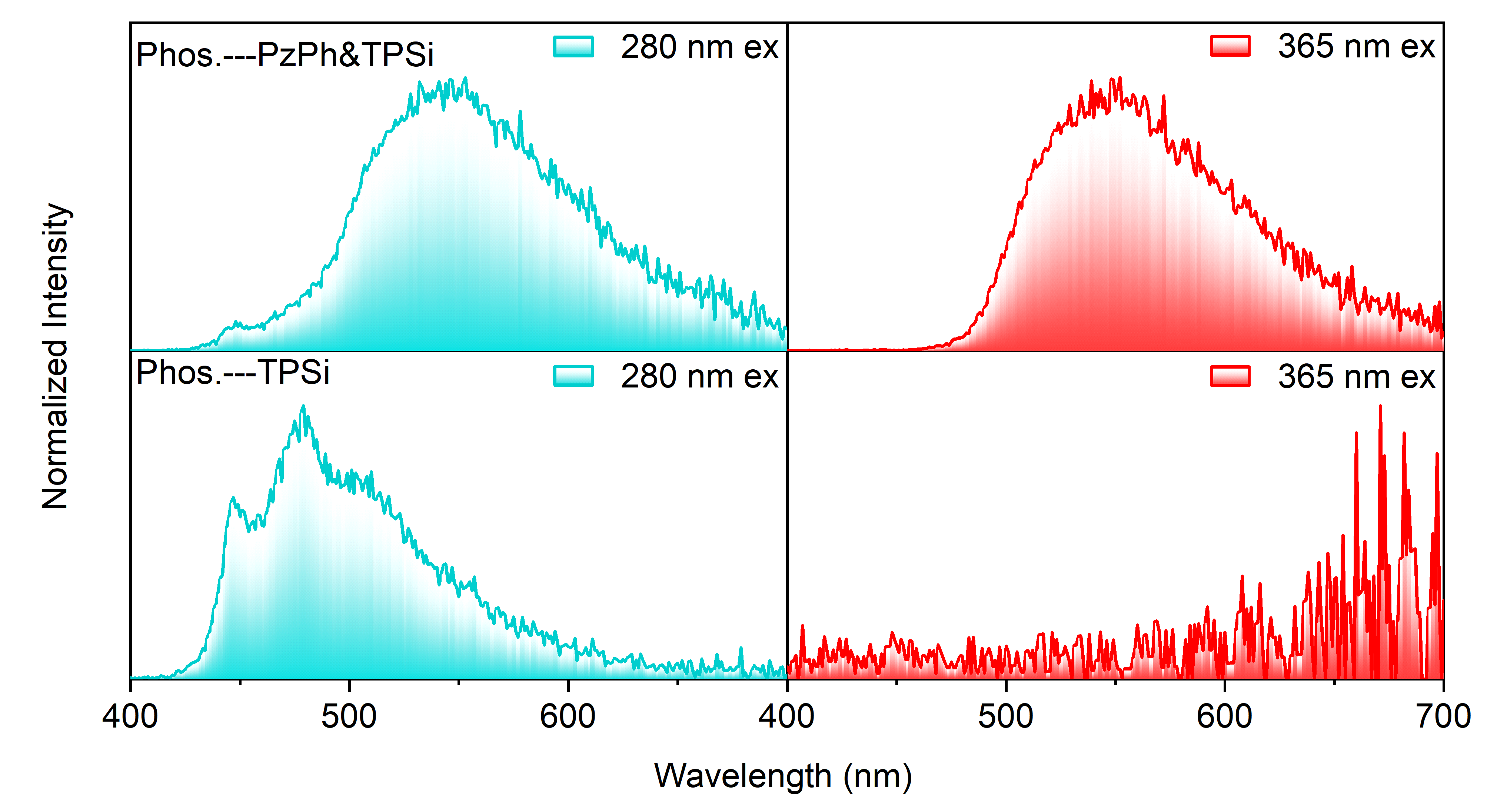


Supplementary Figure 32. Normalized delay emission spectra of PzPh&TPSi and TPSi after the stoppage of different excitation wavelengths (virtual gating from 1 ms to 5 ms).


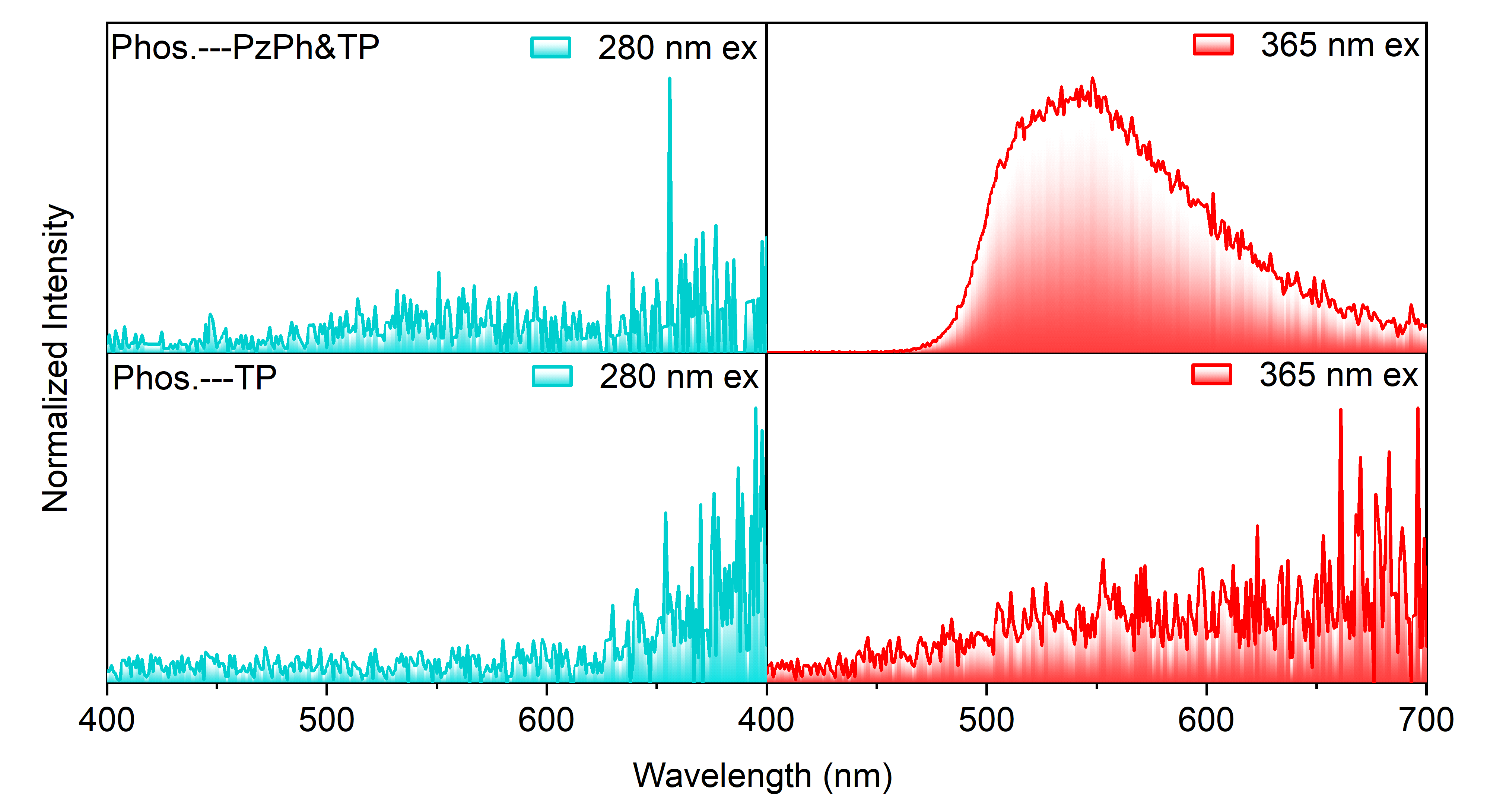


Supplementary Figure 33. Normalized delay emission spectra of PzPh&TP and TP after the stoppage of different excitation wavelengths (virtual gating from 1 ms to 5 ms).


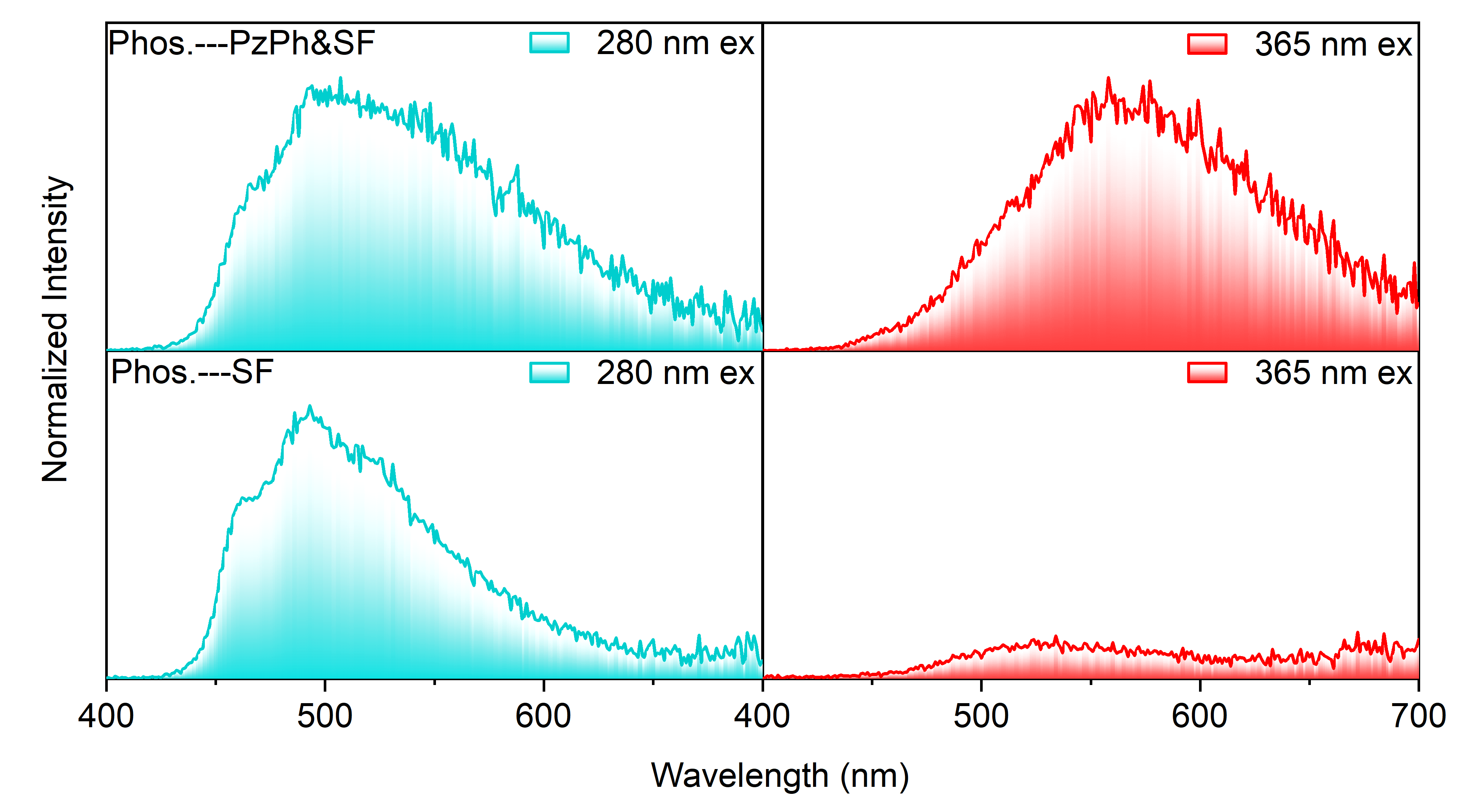


Supplementary Figure 34. Normalized delay emission spectra of PzPh&SF and SF after the stoppage of different excitation wavelengths (virtual gating from 1 ms to 5 ms).


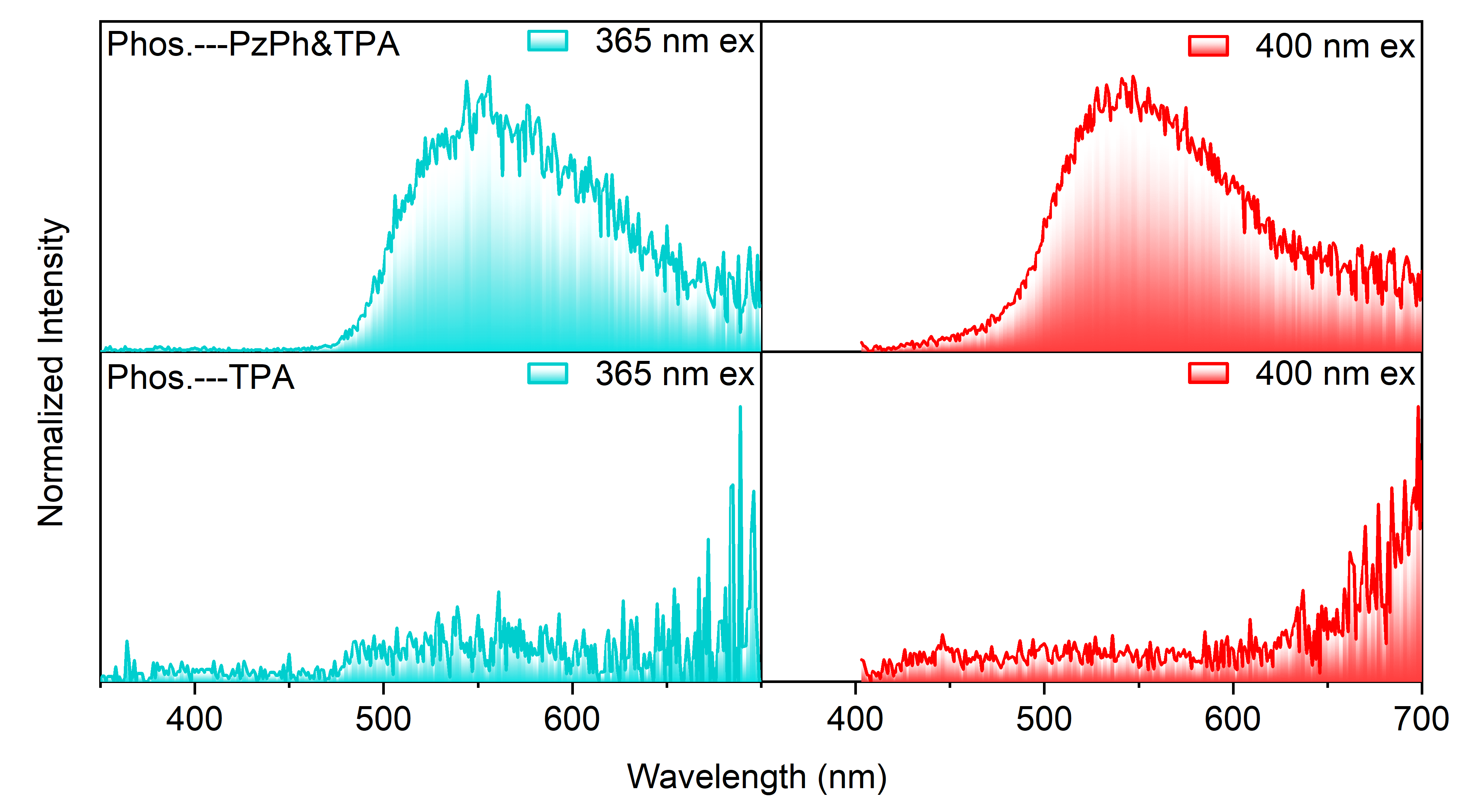


Supplementary Figure 35. Normalized delay emission spectra of PzPh&TPA and TPA after the stoppage of different excitation wavelengths (virtual gating from 1 ms to 5 ms).


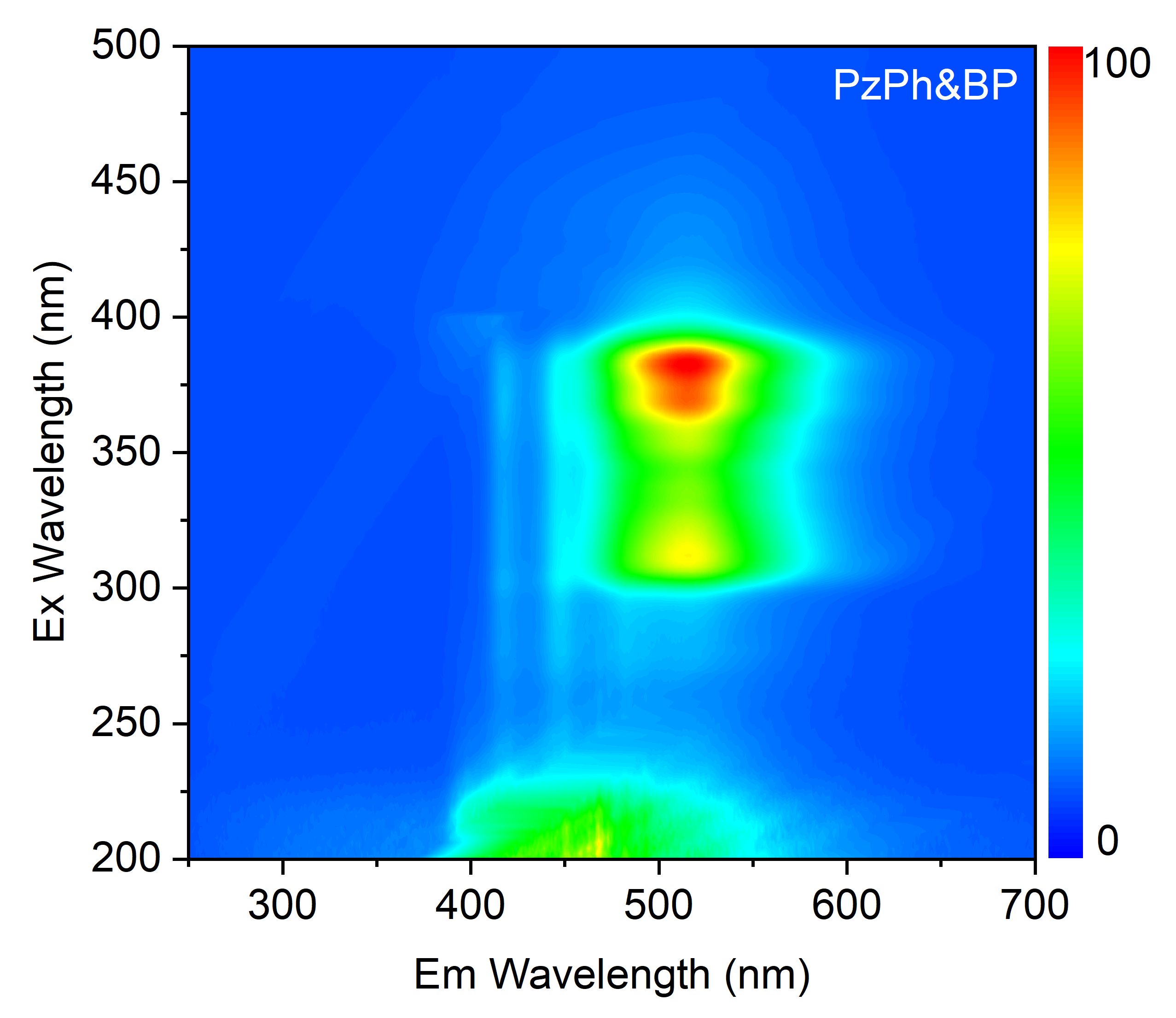


Supplementary Figure 36. Excitation-emission mapping of PzPh&BP in ambient conditions (the Rayleigh scattering was removed by Delaunay triangulation method).


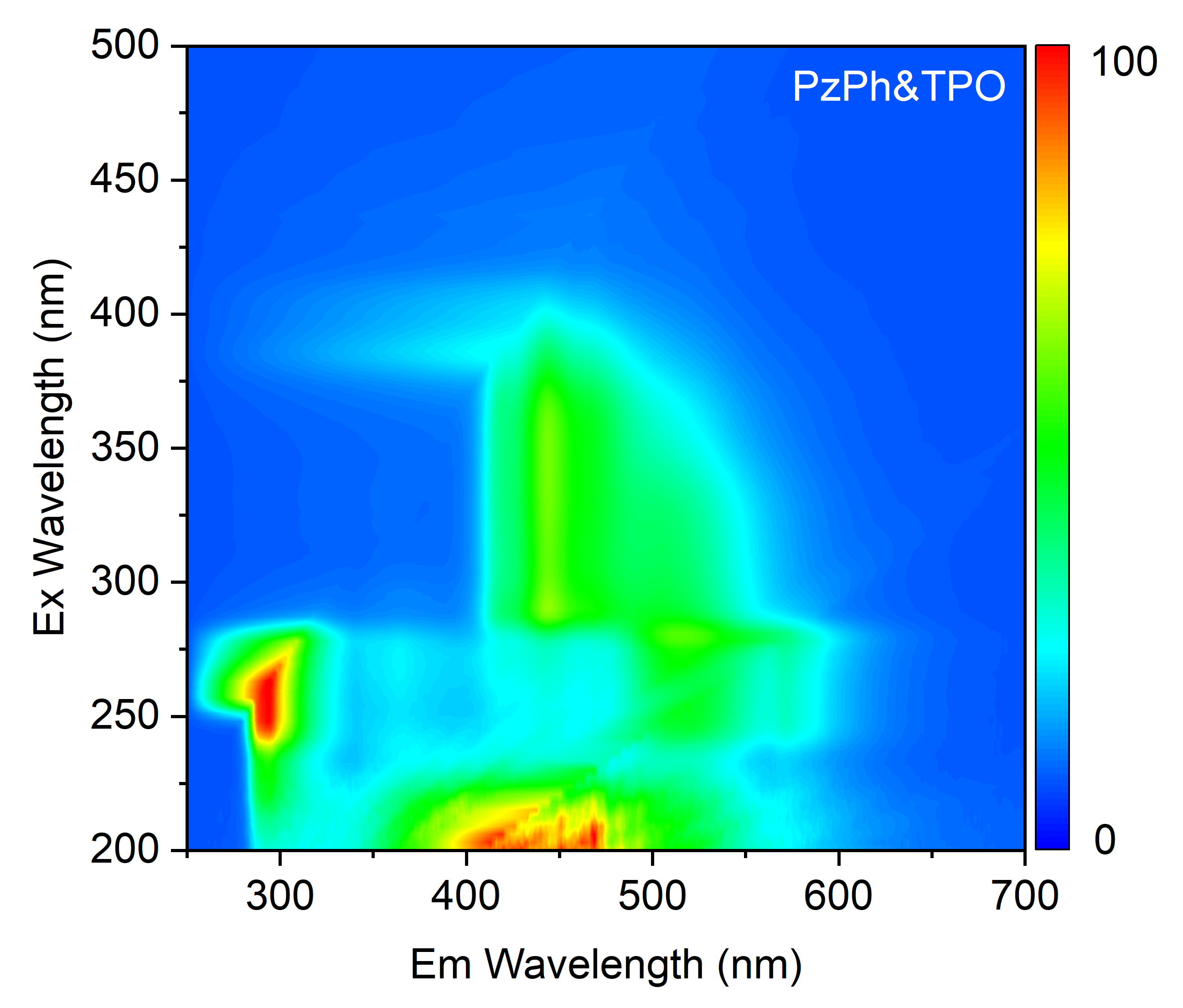


Supplementary Figure 37. Excitation-emission mapping of PzPh&TPO in ambient conditions (the Rayleigh scattering was removed by Delaunay triangulation method).


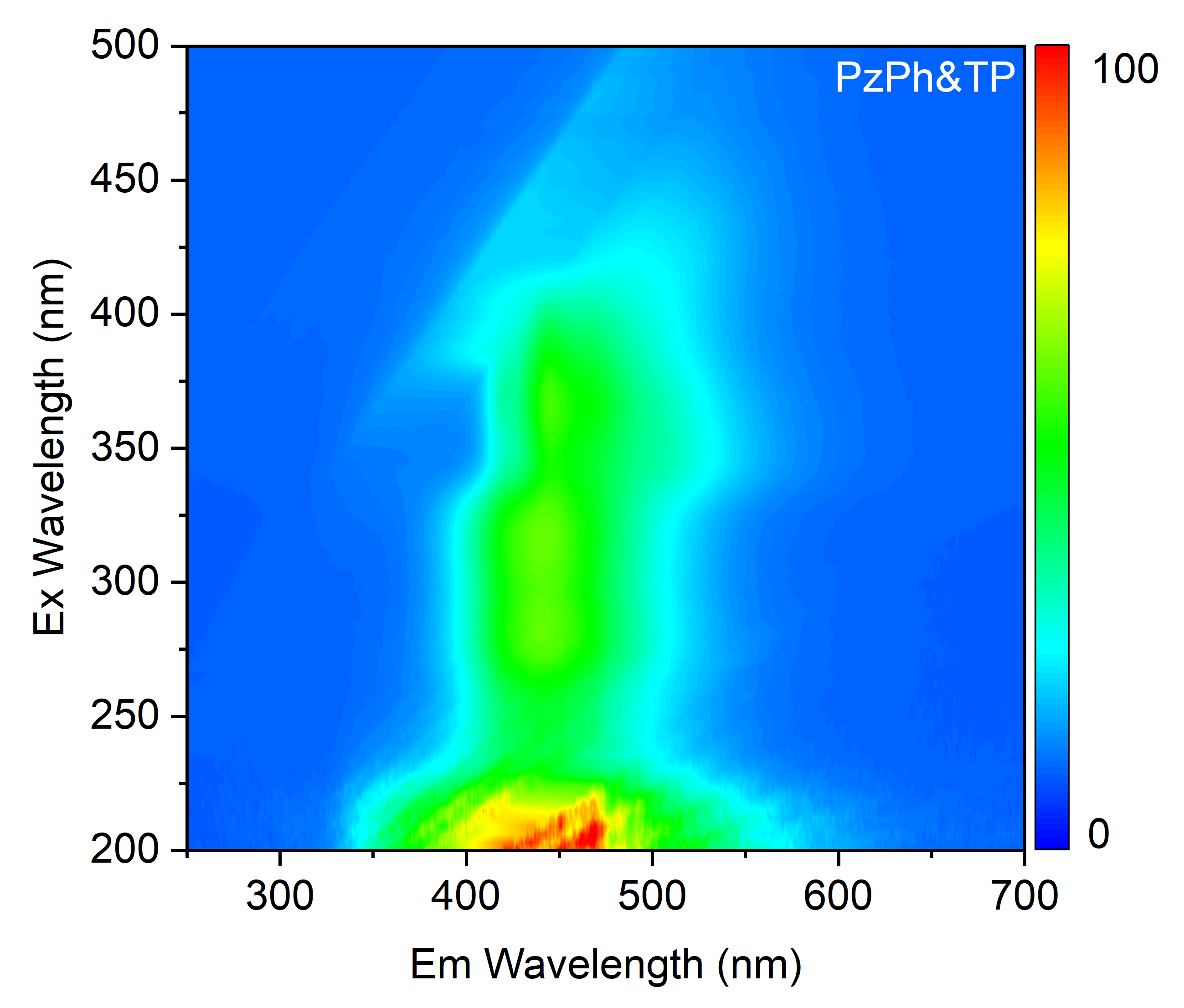


Supplementary Figure 38. Excitation-emission mapping of PzPh&TP in ambient conditions (the Rayleigh scattering was removed by Delaunay triangulation method).


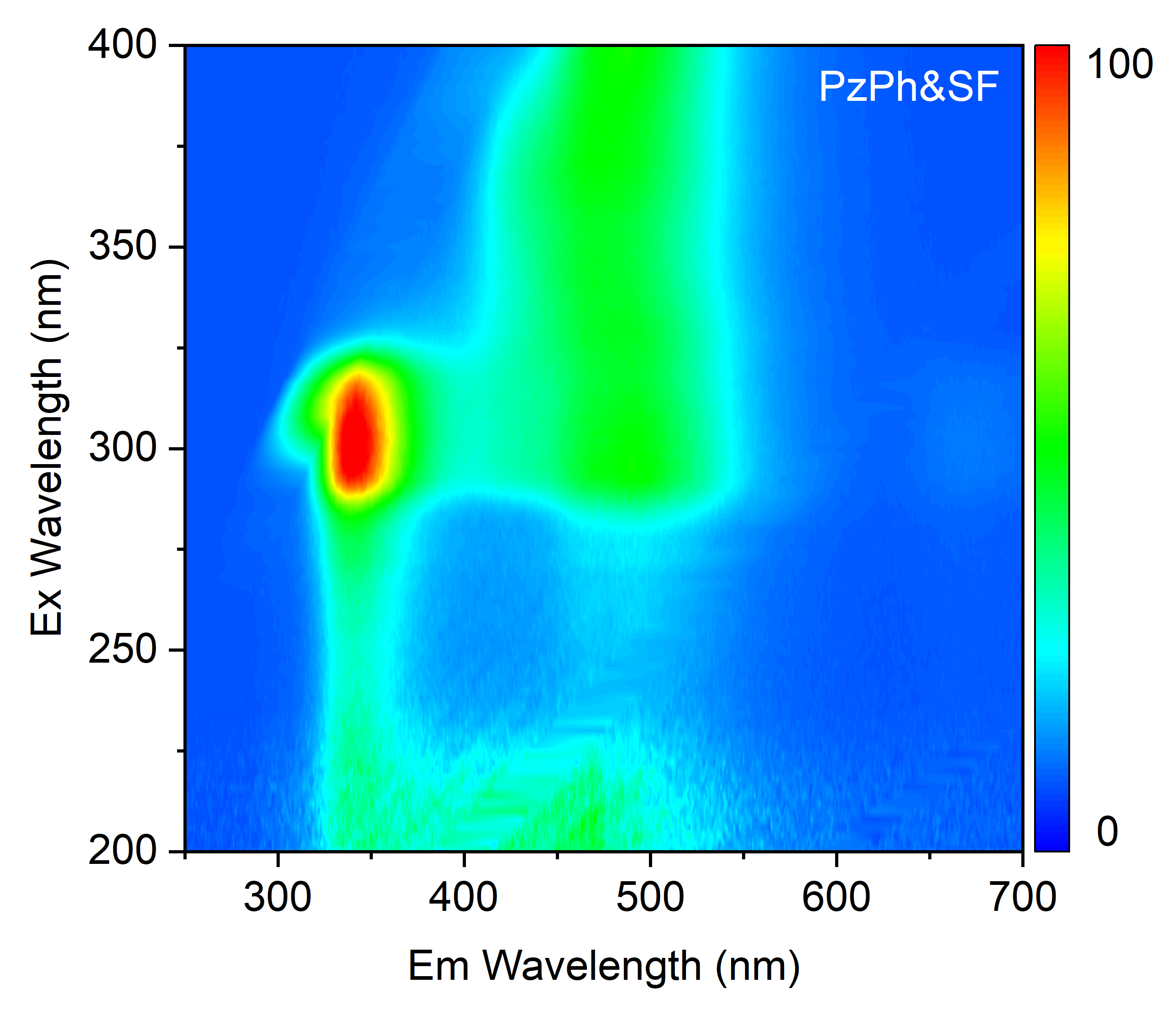


Supplementary Figure 39. Excitation-emission mapping of PzPh&SF in ambient conditions (the Rayleigh scattering was removed by Delaunay triangulation method).


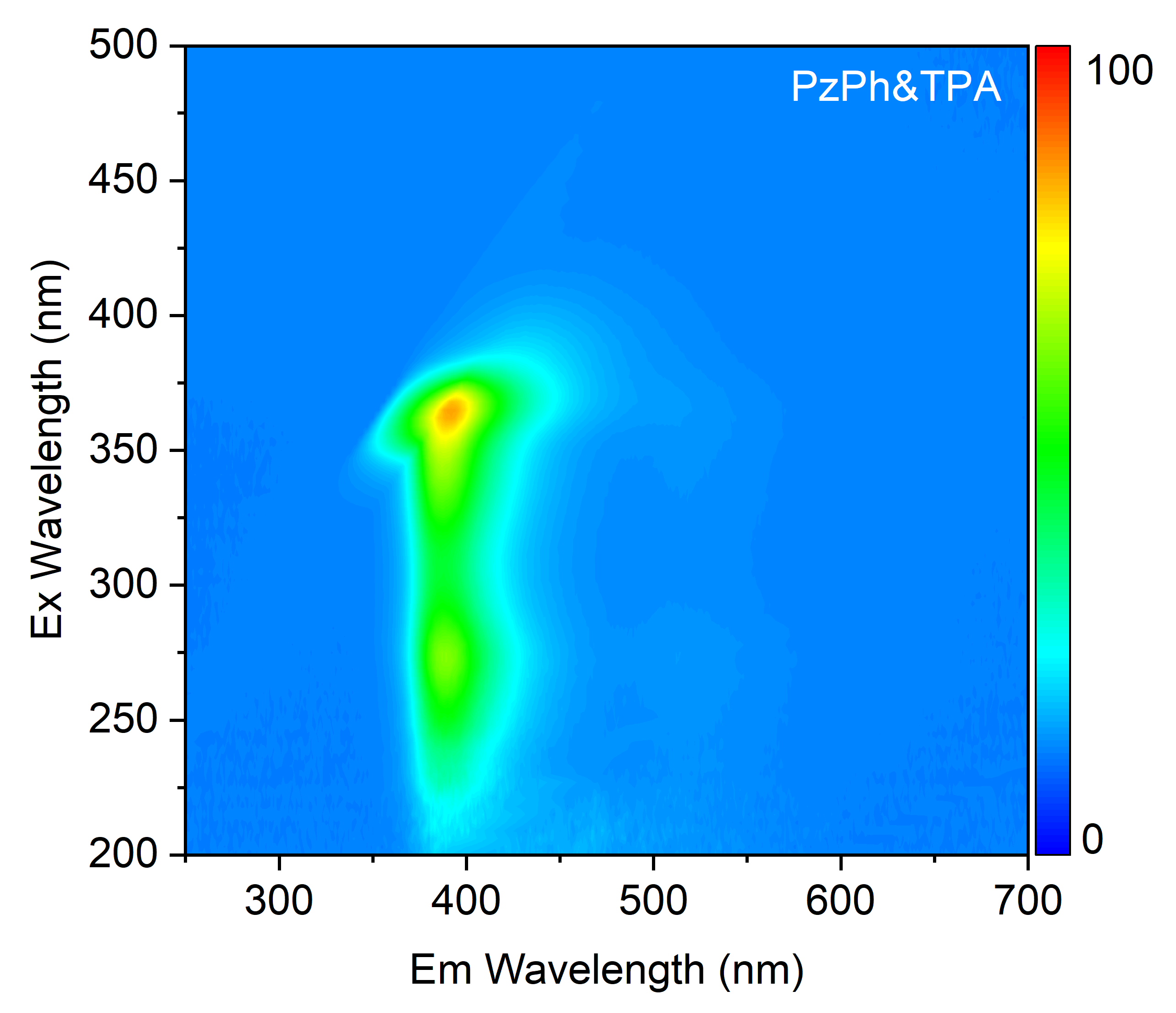


Supplementary Figure 40. Excitation-emission mapping of PzPh&TPA in ambient conditions (the Rayleigh scattering was removed by Delaunay triangulation method).


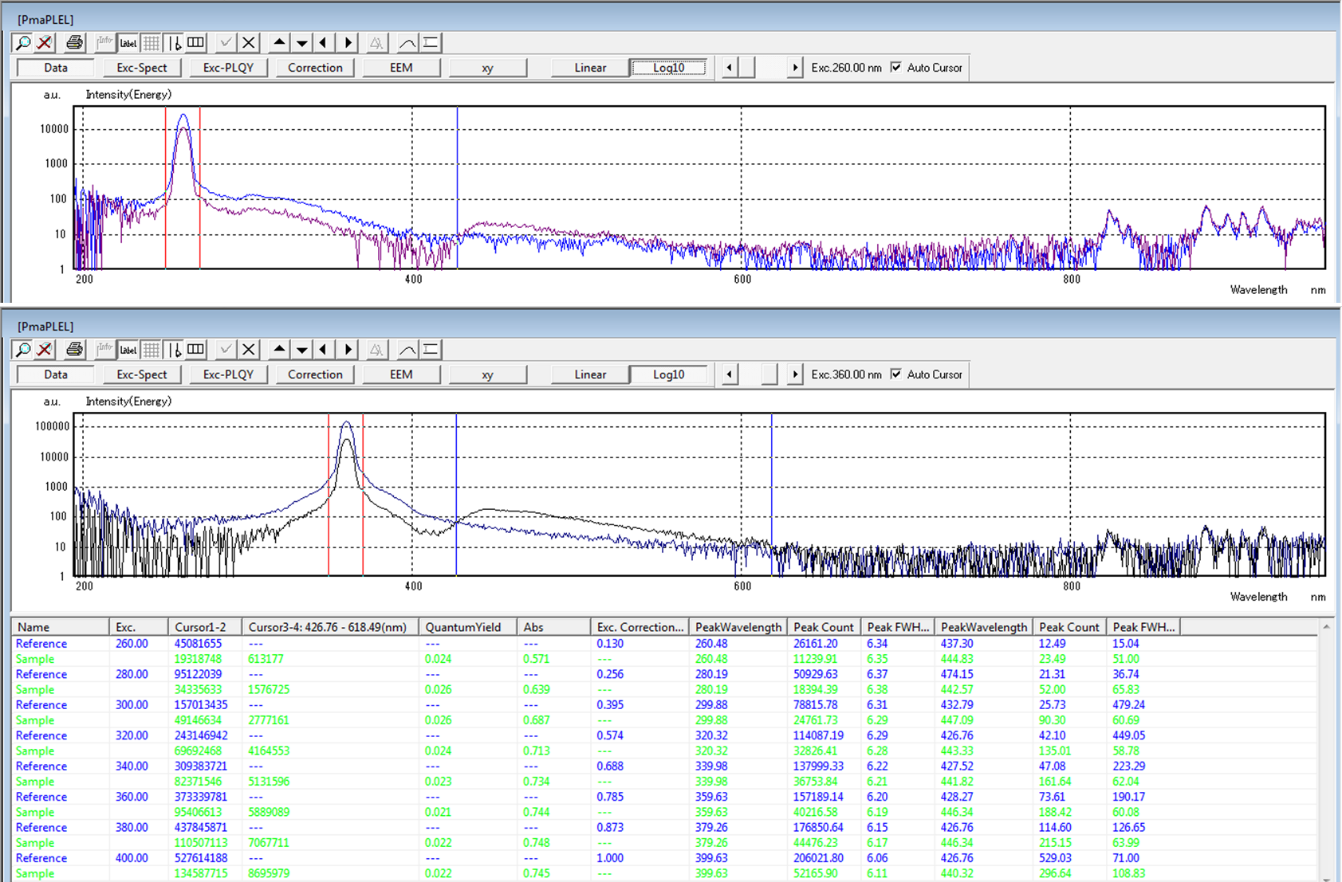


Supplementary Figure 41. Quantum efficiency of the photoluminescence for PzPh under different wavelengths.


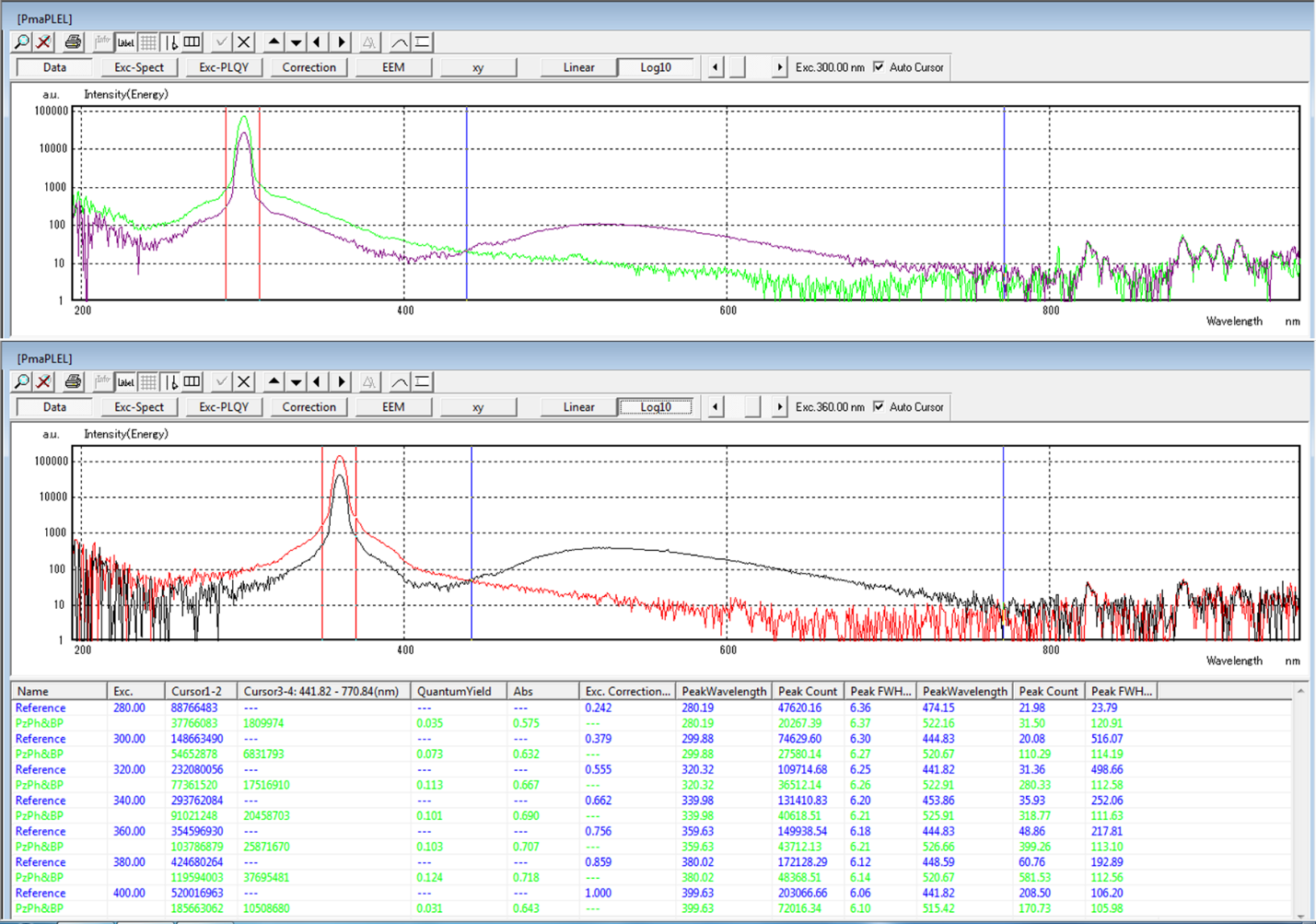


Supplementary Figure 42. Quantum efficiency of the photoluminescence for PzPh&BP under different wavelengths.


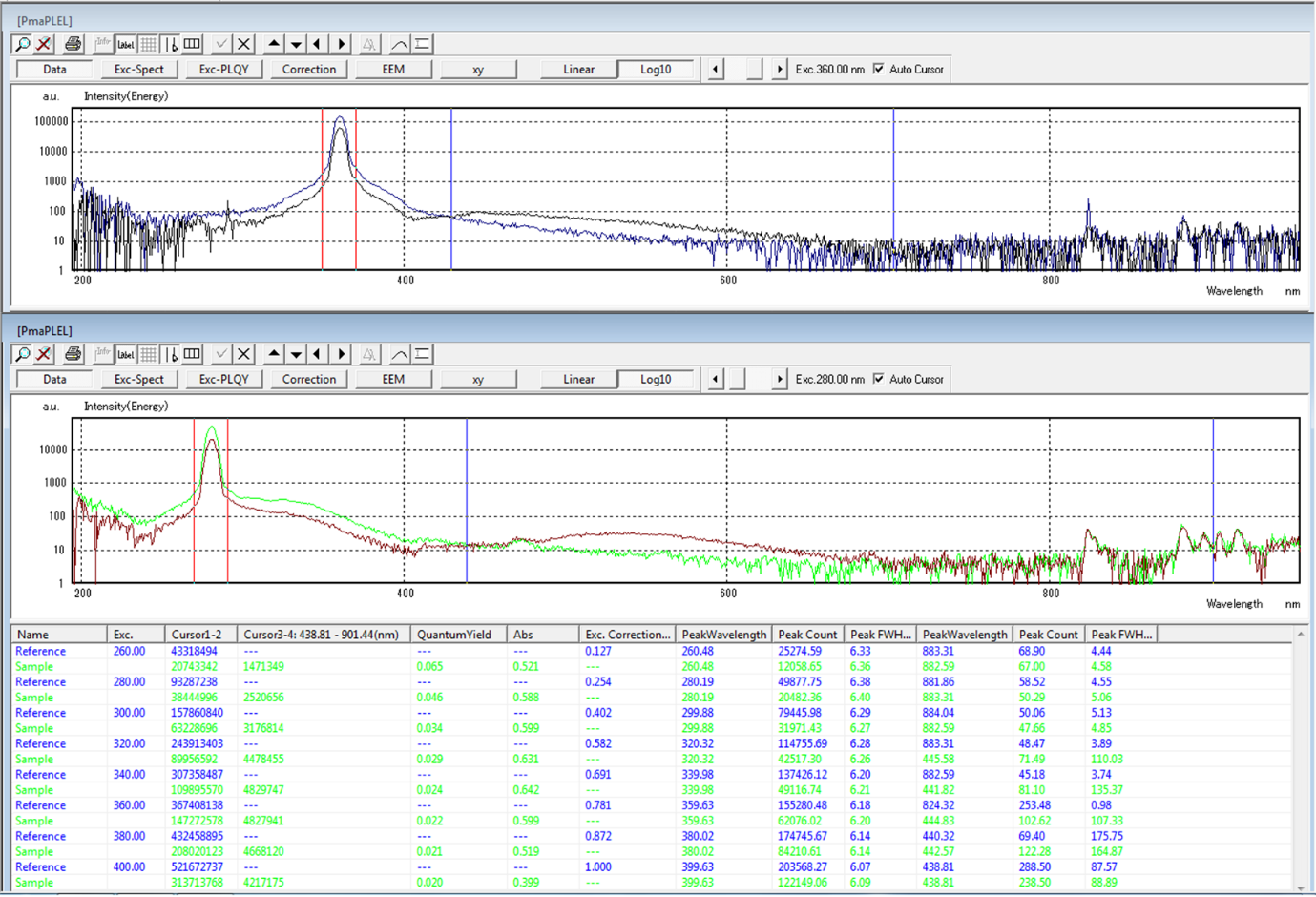


Supplementary Figure 43. Quantum efficiency of the photoluminescence for PzPh&TPO under different wavelengths.


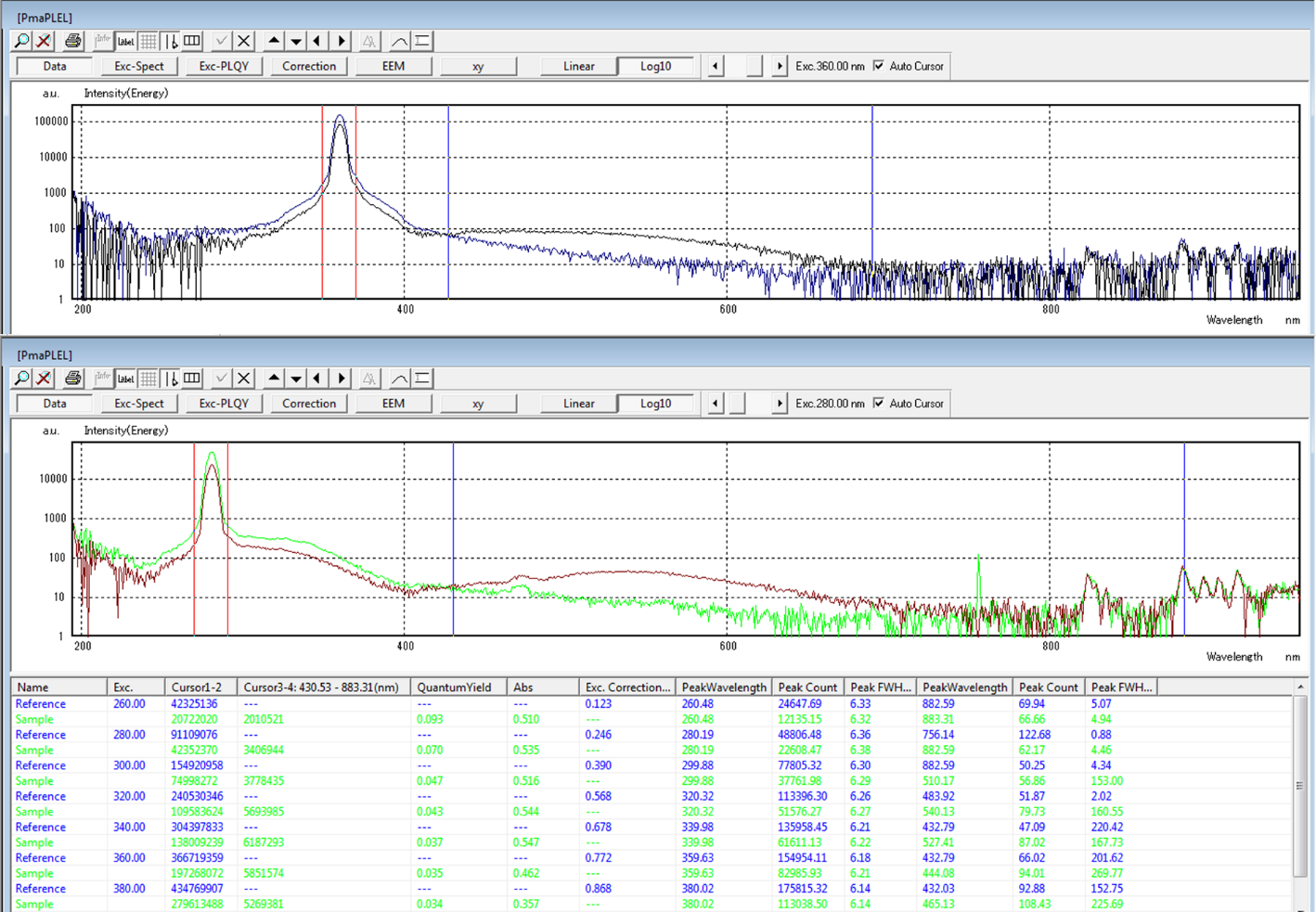


Supplementary Figure 44. Quantum efficiency of the photoluminescence for PzPh&TPSi under different wavelengths.


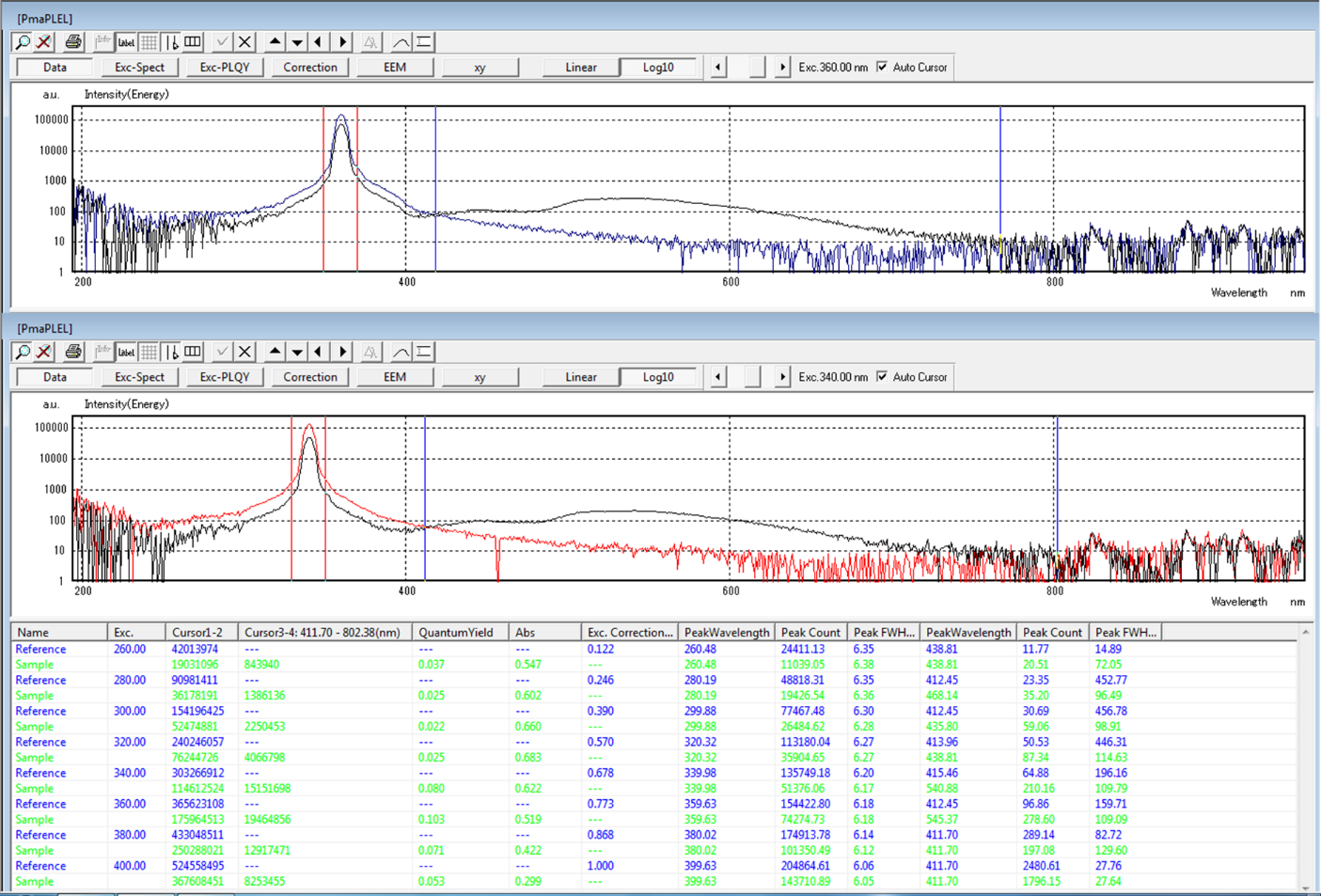


Supplementary Figure 45. Quantum efficiency of the photoluminescence for PzPh&TP under different wavelengths.


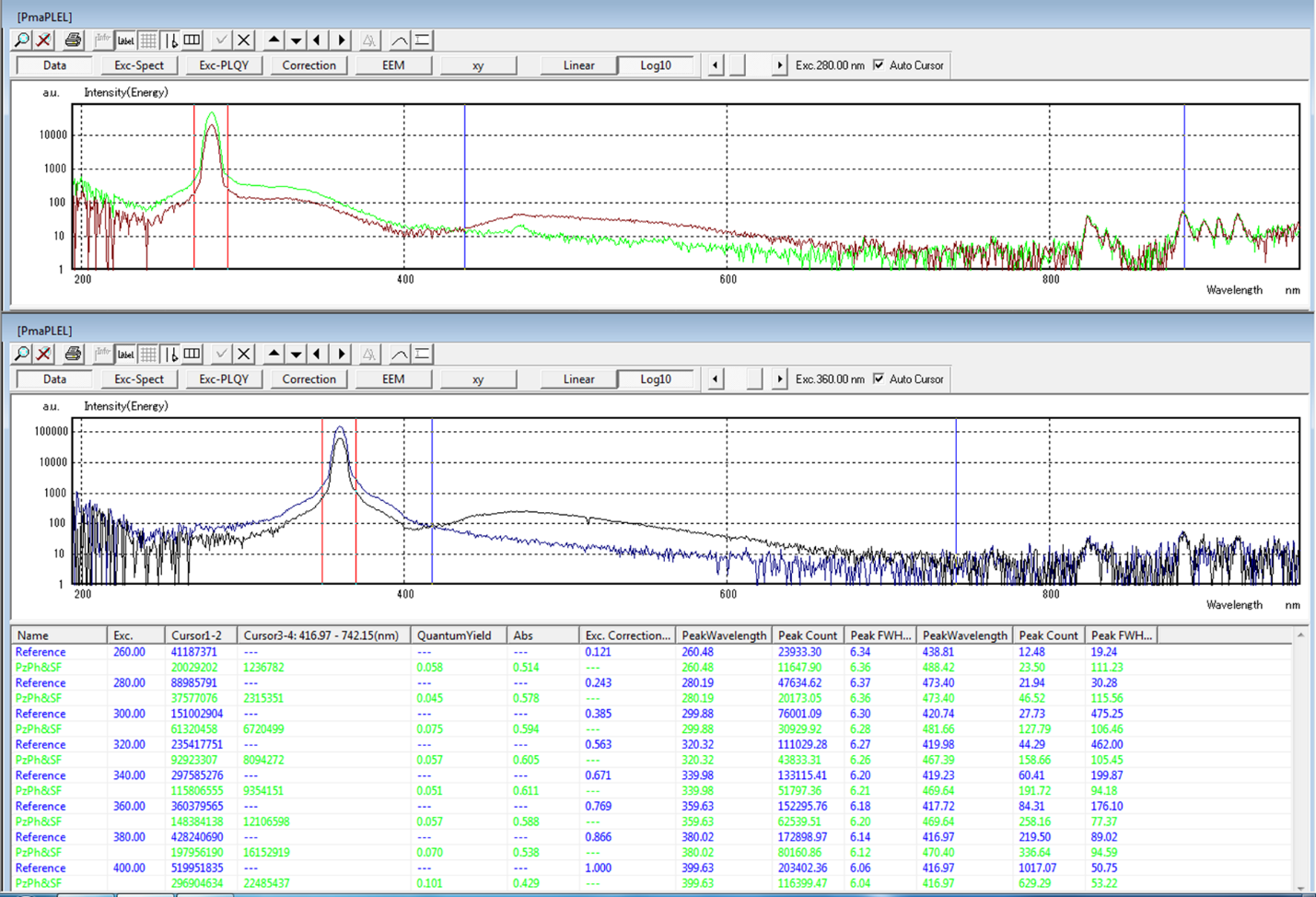


Supplementary Figure 46. Quantum efficiency of the photoluminescence for PzPh&SF under different wavelengths.


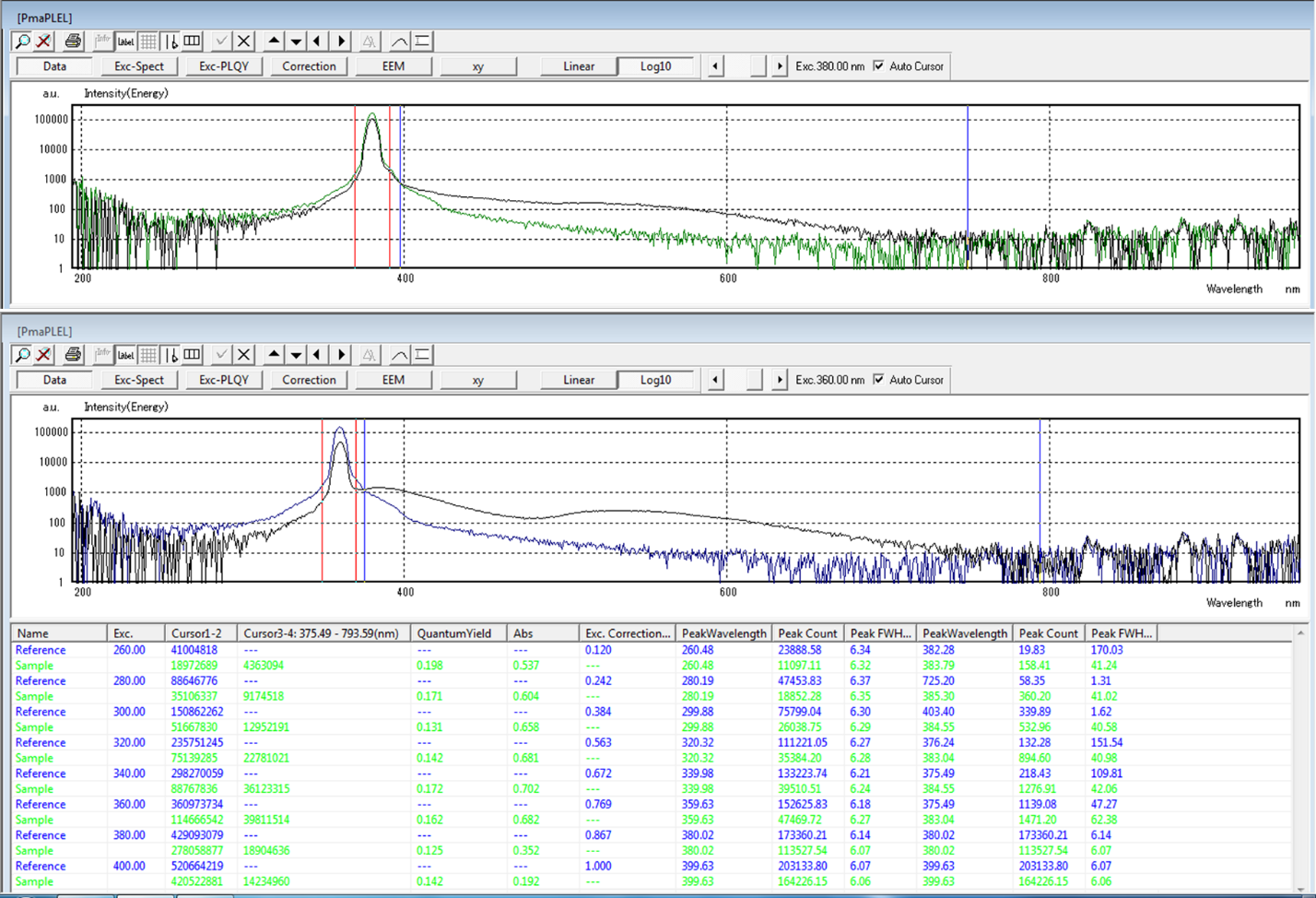


Supplementary Figure 47. Quantum efficiency of the photoluminescence for PzPh&TPA under different wavelengths.


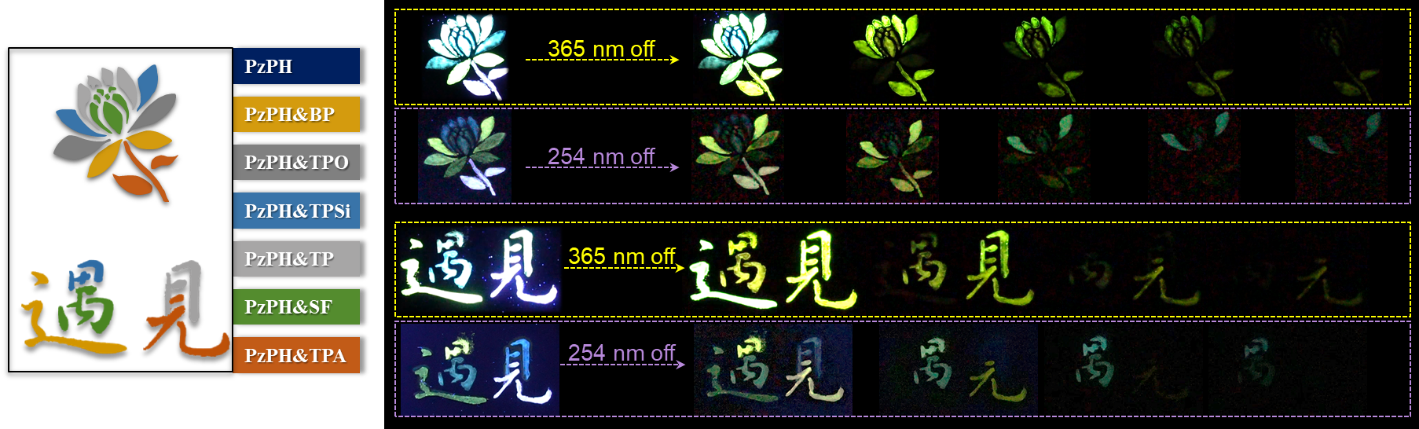


Supplementary Figure 48. Schematic illustrations of lifetime-order encoding property for mH/G UOP materials under different excitation wavelengths at room temperature.


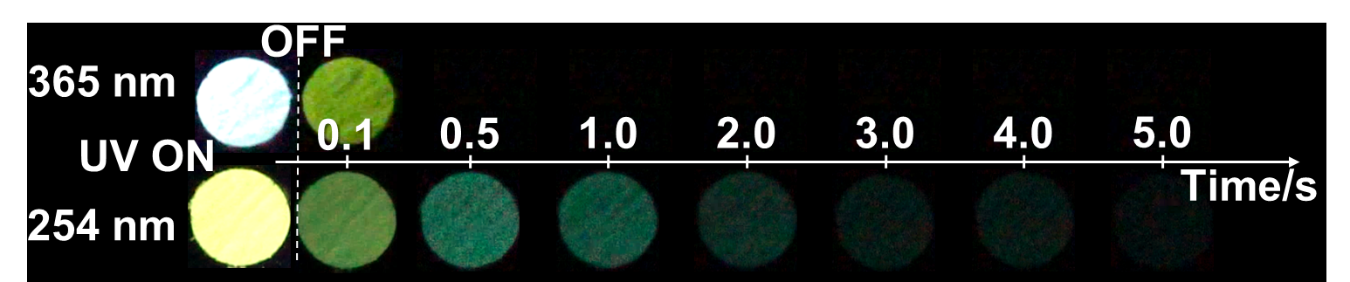


Supplementary Figure 49. Photographs of PzPh&TPSi taken under a 365 or 254 nm UV lamp on and off.

**Supplementary Table**

Supplementary Table 1. Photophysical properties of crystalline powders for PzPh and the mH/G UOP materials.

| **Sample** | ***λ*_ex_ (nm)** | **Fluo.** | | | **Phos.** | | | | |
| --- | --- | --- | --- | --- | --- | --- | --- | --- | --- |
|  |  | ***λ*_F_ (nm)** | ***Φ*_F_ (%)** | ***τ*_F_ (ns)** | ***λ*_P_ (nm)** | ***Φ*_P_ (%)** | ***τ*_P1_ (ms)** | ***τ*_P2_ (ms)** | ***τ*_P3_ (ms)** |
| PzPh | 365 | 445; 472 | 2.1 | 3.7 | 534; 577 | - | - | - | - |
| PzPh&BP | 365 | 445 | 0.9 | 3.7(445 nm) | 543 | 9.4 | 10.6 (0.8%) | 3.9 (99.2%) | - |
|  | 280 | 445 | - | - | 543 | - | 10.2 (3.4%) | 4.8 (96.6%) | - |
| PzPh&TPO | 365 | 445; 477 | 1.1 | 3.8(445 nm) | 547 | 1.3 | 10.3 (29.3%) | 21.5 (70.7%) | - |
|  | 280 | 292; 445 | - | - | 547 | - | 10.6 (19.3%) | 20.5 (72.4%) | 257.0 (8.2%) |
| PzPh&TPSi | 365 | 445; 472 | 0.9 | 3.7(445 nm) | 549 | 2.4 | 11.9 (79.3%) | 30.4 (20.7%) | - |
|  | 280 | 305; 451 | - | - | 450; 549 | - | 11.3 (32.6%) | 31.6 (8.7%) | 778.9 (58.8%) |
| PzPh&TP | 365 | 445; 475 | 1.3 | 3.0(445 nm) | 547 | 8.9 | 23.3 (89.8%) | 43.1 (10.2%) | - |
|  | 280 | 445 | - | - | 547 | - | 22.7 (84.4%) | 43.2 (15.6%) | - |
| PzPh&SF | 365 | 472; 498 | 5.7 | 5.6(445 nm) | 556 | 4.4 | 22.6 (56.9%) | 157.1 (43.1%) | - |
|  | 280 | 341; 492 | - | - | 494; 556 | - | 22.5 (6.7%) | 154.4 (5.2%) | 836.2 (88.1%) |
| PzPh&TPA | 400 | 445;477 | 9.3 | 3.0(445 nm) | 555 | 6.9 | 25.4 (65.8%) | 376.9 (34.2%) | - |
|  | 365 | 390 | - | - | 400; 555 | - | 25.0 (70.0%) | 428.1 (20.8%) | 83.3 (9.2%) |

* The fluorescence and phosphorescence quantum yields of the mH/G UOP system could be calculated separately from the total luminescence quantum yields according to the method in previous literatures.^1, 2^

Supplementary Table 2. photophysical properties of crystalline powders for host materials.

|  | BP | TPO | TPSi | TP | SF | TPA |
| --- | --- | --- | --- | --- | --- | --- |
| *λ_Fluo._* (nm) | **-** | 293 | 318 | - | 337 | 391 |
| *λ_Phos._* (nm) | 446 | 482 | 484 | 531 | 525 | 550 |
| *τ*_P_ (ms) | 1.2 | 183.3 | 848.3 | - | 748.8(77.5%) 202.1(22.5%) | 281.8 |
| *E*_T1_ (*eV*) | 3.19 | 3.07 | 3.04 | 2.90 | 2.83 | 2.66 |
| Space group | *P*212121 | *P*21/c | *P*-421c | *P*-1 | *P*21/a | *C*c |

Supplementary Table 3. Thermal properties of crystalline powders for host materials and PzPh.

| **Sample** | BP | TPO | TPSi | TP | SF | TPA | PzPh |
| --- | --- | --- | --- | --- | --- | --- | --- |
| T_m_ (^o^C) | 52.3 | 159.5 | 241.4 | 83.1 | 127.6 | 128.5 | 97.1 |
| T_d_ (^o^C) | 115.7 | 233.6 | 246.0 | 176.9 | 201.7 | 175.0 | 203.6 |

* melting temperature (Tm) and decomposition temperature (Td) of 5% weight loss under N_2_ atmosphere at 10 ^o^C/min (DSC) and 20 ^o^C/min (TGA) heating rate.

**Supplementary References**

1. Yang, Z. *et al.* Boosting quantum efficiency of ultralong organic phosphorescence up to 52% via intramolecular halogen bonding. *Angew. Chem. Int. Ed.* ***59****,* 17451-17455*,* (2020).
2. Lei, Y. *et al.* Wide-range color-tunable ultralong organic phosphorescence materials for printable and writable security inks. *Angew. Chem. Int. Ed.* ***59***, 16054-16060, (2020).
