# Supplementary material for: Wide-range lifetime-tunable and responsive ultralong organic phosphorescent multi-host/guest system
Source: Nat Commun. 2021 Jun 10;12:3522. doi: 10.1038/s41467-021-23742-4 (PMC8192513; doi:10.1038/s41467-021-23742-4)
Supplement: Supplementary file 3 — Description of Additional Supplementary Files [file 41467_2021_23742_MOESM3_ESM.pdf]

Description of Additional Supplementary Files

File Name: Supplementary Movie 1.
Description: The video with 0.1 times speed shows the afterglows of the mH/G UOP system along with duration time after the stoppage of 365 nm UV excitation, which adopts the pattern of Fig. 4a-3 and is corresponding to Fig. 4d. By taking advantages of the characteristics of distinguishable time-resolved triplet excitons decay, the afterglow with “B8”, “A9”, “P3”, “F3”, “^{C}7” and “^{L}1” exhibited within 1 s in a simply fabricated thin-film.

File Name: Supplementary Movie 2.
Description: The video with 0.1 times speed shows the afterglows of the mH/G UOP system along with duration time after the stoppage of 365 nm UV excitation, which adopts the pattern of Fig. 4a-1 and is corresponding to Fig. 4c. It shows the order that the birds fade away before the branch after cease of 365 nm UV excitation.

File Name: Supplementary Movie 3.
Description: The video with 0.1 times speed shows the afterglows of the mH/G UOP system along with duration time after the stoppage of 254 nm UV excitation, which adopts the pattern of Fig. 4a-1 and is corresponding to Fig. 4c. In contrast to the Supplementary Movie 2, the pattern displays different dynamic information (UOP lifetime and color) after cease of 254 nm UV light irradiation. It shows the order that the branch fades away between the two birds.

File Name: Supplementary Movie 4.
Description: The video with 0.1 times speed shows the afterglow bar code of the mH/G UOP system along with duration time after the stoppage of 365 nm UV excitation, which adopts the pattern of Fig. 4a-4 and is corresponding to Fig. 4e. In this video, the afterglow bar code varies with duration time of the UOP. The encrypted information “IFE” could only be identified at about 200 ms (before reading the information, desaturated and reversed-phase processes should be carried out).
